# Nectary development in *Cleome violacea*

**DOI:** 10.3389/fpls.2022.1085900

**Published:** 2023-02-09

**Authors:** Shane Carey, Brandi Zenchyzen, A. J. Deneka, Jocelyn C. Hall

**Affiliations:** Department of Biological Sciences, University of Alberta, Edmonton, AB, Canada

**Keywords:** Cleomaceae, nectaries, nectar, transcriptomics, VIGS, parallel evolution, RNA-seq

## Abstract

Nectaries are a promising frontier for plant evo-devo research, and are particularly fascinating given their diversity in form, position, and secretion methods across angiosperms. Emerging model systems permit investigations of the molecular basis for nectary development and nectar secretion across a range of taxa, which addresses fundamental questions about underlying parallelisms and convergence. Herein, we explore nectary development and nectar secretion in the emerging model taxa, *Cleome violacea* (Cleomaceae), which exhibits a prominent adaxial nectary. First, we characterized nectary anatomy and quantified nectar secretion to establish a foundation for quantitative and functional gene experiments. Next, we leveraged RNA-seq to establish gene expression profiles of nectaries across three key stages of development: pre-anthesis, anthesis, and post-fertilization. We then performed functional studies on five genes that were putatively involved in nectary and nectar formation: *CvCRABSCLAW* (*CvCRC)*, *CvAGAMOUS* (*CvAG), CvSHATTERPROOF (CvSHP), CvSWEET9*, and a highly expressed but uncharacterized transcript. These experiments revealed a high degree of functional convergence to homologues from other core Eudicots, especially *Arabidopsis*. *CvCRC*, redundantly with *CvAG* and *CvSHP*, are required for nectary initiation. Concordantly, *CvSWEET9* is essential for nectar formation and secretion, which indicates that the process is eccrine based in *C. violacea*. While demonstration of conservation is informative to our understanding of nectary evolution, questions remain. For example, it is unknown which genes are downstream of the developmental initiators *CvCRC*, *CvAG*, and *CvSHP*, or what role the *TCP* gene family plays in nectary initiation in this family. Further to this, we have initiated a characterization of associations between nectaries, yeast, and bacteria, but more research is required beyond establishing their presence. *Cleome violacea* is an excellent model for continued research into nectary development because of its conspicuous nectaries, short generation time, and close taxonomic distance to *Arabidopsis*.

## 1 Introduction

Flowers exhibit tremendous diversity of form, much of which is driven by plant-pollinator interactions. Responses to similar pollinator environments have resulted in repeated evolution of floral forms across angiosperms (reviewed in: (Endress, 2011; Sauquet et al., 2017; Wessinger and Hileman, 2020)). Such traits include, but are not limited to, monosymmetry (zygomorphy), organ fusion, spurs, and heterostyly (Specht and Howarth, 2015; Kramer, 2019; Phillips et al., 2020; Wessinger and Hileman, 2020). This repeated evolution raises fundamental questions about the developmental and genetic bases of their evolutionary shifts (Sobel and Streisfeld, 2013; Specht and Howarth, 2015; Kramer, 2019; Wessinger and Hileman, 2020). Among these questions is whether the same genetic pathways have been recruited in independent origins of these traits (Specht and Howarth, 2015; Wessinger and Hileman, 2020). Remarkable and repeated recruitment of the same genetic pathway is clear with certain traits, notably monosymmetry [reviewed in: (Preston et al., 2009; Preston et al., 2011; Hileman, 2014a; Hileman, 2014b; Wessinger and Hileman, 2020)], but whether the genetic basis of other features is conserved remains unclear.

Nectaries, and the nectar they secrete, are integral to plant-animal interactions and, as such, warrant detailed investigation across taxa (Liao et al., 2021). They have evolved multiple times across angiosperms and are remarkably variable in position, structure, and morphology (Bernardello, 2007; Nepi, 2007; Nepi et al., 2018; Liao et al., 2021; Slavkovic et al., 2021). Despite this variation, nectaries are united by the simple function of producing nectar, a complex sugar-rich solution that contains a wide range of metabolites and microbes (Nepi, 2007; Heil, 2011; Nepi et al., 2018; Slavkovic et al., 2021; Liao et al., 2021). As a critical reward to insects, and potential attractor, nectaries and their nectar drive many macroevolutionary patterns *via* relationships with pollinators and other animals (Parachnowitsch et al., 2019; Liao et al., 2021). Nectaries are associated with all plant organs except for roots, and floral nectaries can be associated with any floral organ (Nepi, 2007; Liao et al., 2021). Nectary morphology can be structured (i.e., distinct morphology with identifiable cell types) or unstructured (i.e., no specialized morphology) (Nepi, 2007; Slavkovic et al., 2021). Nectar secretion ranges from modified stomata (nectarostomata), to specialized trichomes, and even cell rupture (reviewed in: (Nepi, 2007; Slavkovic et al., 2021)). This diversity in morphology and secretion mechanisms differs across families and within genera (Bernardello, 2007). Also, variable nectar composition impacts pollinator interactions (Nepi et al., 2018; Parachnowitsch et al., 2019). This extensive diversity calls into question whether nectary development is underpinned by similar or different developmental programs in taxa with variable nectaries.

A genetic breakthrough in nectary research was the establishment of *CRABS CLAW* (*CRC*), a YABBY family transcription factor, as essential for nectary initiation (Alvarez and Smyth, 1998; Bowman and Smyth, 1999; Lee et al., 2005a). In *Arabidopsis*, *CRC* knockouts do not develop nectaries (Bowman and Smyth, 1999). *CRC* has since been shown as essential for nectary formation across the core eudicots (Lee et al., 2005b; Fourquin et al., 2014), and is expressed in extrafloral nectaries (Lee et al., 2005b). *CRC* protein dimerizes with other YABBY transcription factors and also has an important role in *Arabidopsis* carpel development (Alvarez and Smyth, 1998; Alvarez and Smyth, 1999; Alvarez and Smyth, 2002; Lee et al., 2005a) that is widely conserved (Orashakova et al., 2009; Fourquin et al., 2014; Pfannebecker et al., 2017). The expression of *CRC* across core eudicot nectaries, regardless of morphology or position, suggests that *CRC* regulation of nectary development is consistent across the clade (Lee et al., 2005b). To the best of our knowledge, functional studies of nectaries have only been conducted in four core Eudicot taxa: *Petunia* (Solonales) (Lee et al., 2005b; Morel et al., 2018), *Gossypium* (Malvales) (Pei et al., 2021), *Pisum* (Fabales) (Fourquin et al., 2014), and *Arabidopsis* (Brassicales) (Bowman and Smyth, 1999). *CRC* is shown as essential for nectary development in the aforementioned taxa, except for *Gossypium* where the gene *GoNe* is required for both floral and extra floral nectaries (Pei et al., 2021). Thus, investigations of additional taxa are needed to uncover the extent of this potential conserved role of *CRC*.

The role of *CRC* as essential for nectary development does not extend beyond the core eudicots. For example, all petals of *Aquilegia* have elongated spurs, which bear nectaries in their distal tips. In this taxa, three *STYLISH* (*STY*) homologs, a member of the *SHORT INTERNODES* (*SHI*) gene family, are redundantly necessary for the formation of nectaries in the spurs as well as style development (Min et al., 2019). Thus, both *CRC* and *STY* are involved in nectary and gynoecial development (Pfannebecker et al., 2017), which raises questions about developmental pathways shared between nectaries and carpels.

Upstream regulators of *CRC* are also shared between *Petunia* and *Arabidopsis* (Morel et al., 2018). *CRC* is insufficient for ectopic nectary formation (Baum et al., 2001), which reveals a necessity for upstream regulators. In *Arabidopsis*, these regulators include ABC(E) class genes *APETALA2/3* (*AP2/3*), *PISTILLATA* (*PI*), *AGAMOUS* (*AG*), and *SEPALLATA1*/2/3 (*SEP1/2/3*), as well as MADS-box gene *SHATTERPROOF 1/2* (*SHP1/2*) (Reviewed in: (Slavkovic et al., 2021)). In sum, *SHP1*/2 and *AG* act redundantly to promote *CRC*, such that knockouts of each one alone does not prevent nectary formation, although combined they do (Lee et al., 2005a). The floral meristem identity genes *LEAFY* (*LFY*) and *UNUSUAL FLORAL ORGANS* (*UFO*) are also upstream of *CRC* and function to restrict *CRC* expression to nectaries and carpels (Bowman and Smyth, 1999; Slavkovic et al., 2021). Loss of *SEP1/2/3* also prevents nectaries from developing (Lee et al., 2005a). Individual knockouts of any aforementioned gene do not prevent nectary formation, although they can impact shape and size (e.g., *lfy, ufo, pi, ag*) (Baum et al., 2001). Double and triple knockouts however cause a loss of nectaries (e.g., *lfy & ufo, sep1/2/3*) (reviewed in: (Slavkovic et al., 2021)). Nectary inhibition may be indirect because meristem identity genes act upstream of ABC class genes, i.e., nectary formation may be halted because their associated organs fail to form. In *Petunia*, nectary formation is also dependent on C class genes, i.e., *euAG* and *PLEN* are essential for nectary formation (Morel et al., 2018). This redundancy of MADS-box genes implies that the entire regulatory pathway was established prior to the Rosid/Asterid split (Morel et al., 2018; Slavkovic et al., 2021).

Beyond nectary formation, genes have been identified that are important for nectary size and growth. In *Petunia*, two *euAP2* genes, *BLIND ENHANCER* (*BEN*) and *REPRESSOR OF B FUNCTION* (*ROB*), impact floral nectary size such that *rob1 rob2 rob3* triple mutants have flowers with larger nectaries than wildtype (Morel et al., 2018). This phenotype is enhanced when *BEN* is also knocked out, such that much of the carpel is converted to nectary tissue (Morel et al., 2018). Whereas in *Arabidopsis, BLADE ON PETIOLE 1/2* (*BOP1/2*) are essential for nectary growth independent of *CRC* (Mckim et al., 2008). Knockouts of *BOP1/2* result in nectaries that are small and not fully differentiated into parenchyma and secretory tissue (Mckim et al., 2008).

Phytohormones also play an important role in nectary development, composition, and secretion. *AUXIN RESPONSE FACTOR 6/8* (*ARF6/8*) promote and coordinate nectary formation in *Arabidopsis* (Reeves et al., 2012) and *Aquilegia* (Zhang et al., 2020). Thus, while these taxa differ in which key regulator promotes nectary formation, they have a shared response to hormone signalling, which reflects the central role of plant hormones in floral evolution (Wessinger and Hileman, 2020). Auxin plays an additional role in nectar secretion *via PIN FORMED 6* (*PIN6*) expression, which is positively correlated with nectar production (Bender et al., 2013). Also correlated with an increase in nectar production is jasmonic acid (JA), which peaks in concentration just prior to nectar secretion in *Brassica napus* (Radhika et al., 2010). Further, both auxin and JA are regulated by gibberellic acid (GA) (Reeves et al., 2012), which speaks to the complex interplay between auxin, JA and GA.

**Figure 1:**
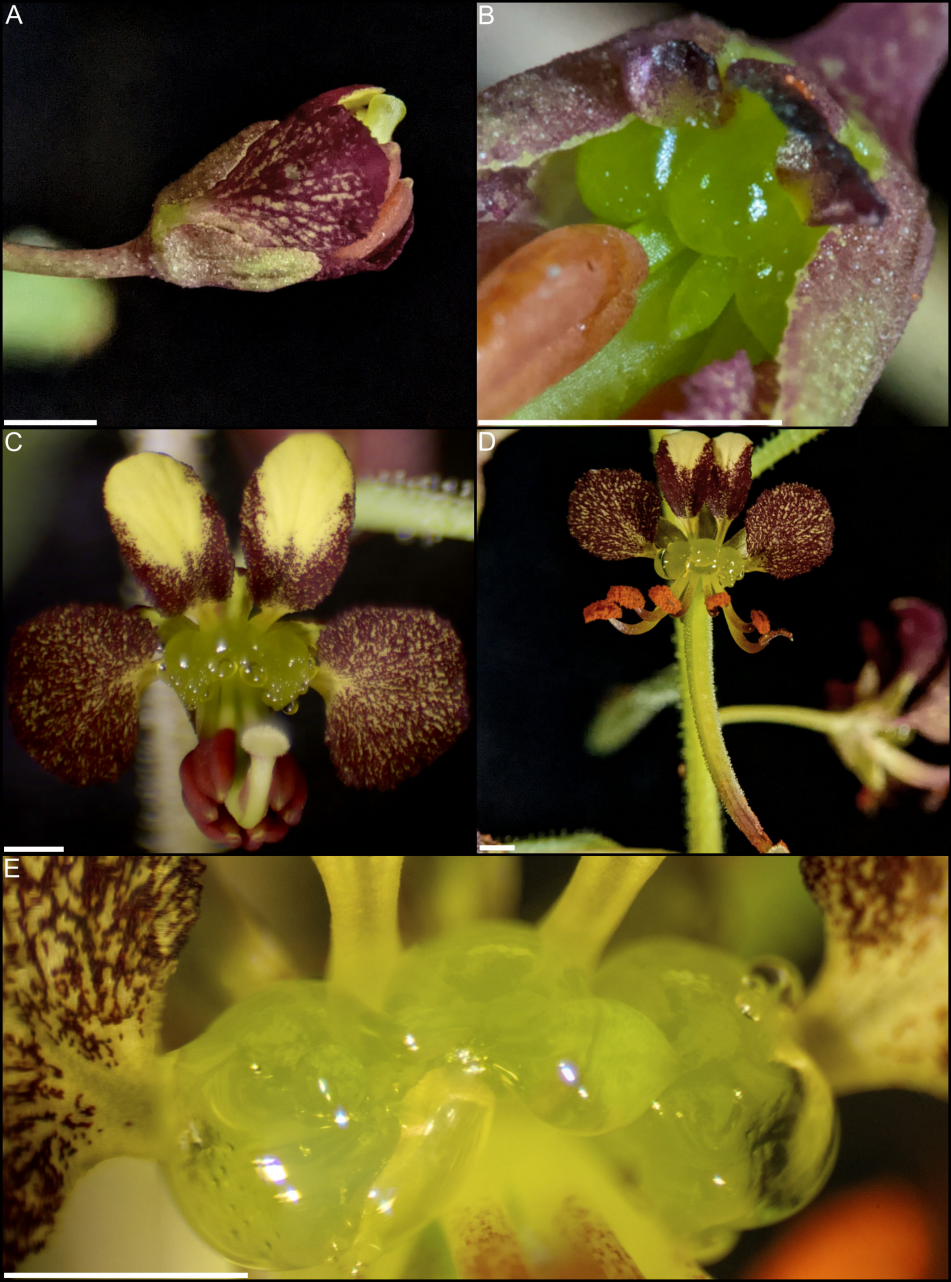
*Cleome violacea* flowers at various stages of development. **(A)** Large undissected floral bud. **(B)** Large dissected floral bud showing nectary. **(C)** Newly anthetic flower. **(D)** Post-anthetic flower with developing fruit. **(E)** Magnified view of anthetic nectary. Scale bars = 1 mm.

Investigations of additional taxa are critical for assessing not only the extent of the conserved role of *CRC*, but also how it is regulated, and the potential pathway deviations across taxa with different nectary shapes and positions. Towards addressing these outstanding questions, Cleomaceae is an excellent model for investigating floral development. Cleomaceae is a small, cosmopolitan family of circa 270 species placed in 25 genera (Bayat et al., 2018). This family houses floral variation in traits likely associated with pollinator interactions, including petal color, petal size, and gynophores/androgynophores (Iltis et al., 2011; Higuera-Diaz et al., 2015; Bayat et al., 2018). Importantly, members of the family exhibit a wide range of nectary size, shape, and position. Across the family, nectaries may be absent, adaxially positioned, or annular (Tucker and Vanderpool, 2010). Cleomaceae is sister to Brassicaceae and the phylogenetic framework within the family is established (Patchell et al., 2014; Barrett et al., 2017; Bayat et al., 2018). While some floral developmental patterns are described (Erbar and Leins, 1997b; Erbar and Leins, 1997a; Patchell et al., 2011), most information regarding nectaries is based on floristic work (e.g., (Tucker and Vanderpool, 2010)). There is also limited empirical information on pollinators, which has revealed generalist and specialist systems across the family (Cane, 2008; Fleming et al., 2009; Higuera-Diaz et al., 2015; Raju and Rani, 2016). Of note, functional approaches have been established for *Cleome violacea* (Carey et al., 2021). This species is amenable to investigations of nectaries as their flowers have prominent, 3-lobed nectaries adaxially positioned between petals and stamen (**Figure 1**).

The overarching goal of this study was to determine the genetic basis of floral nectaries of *Cleome violacea*. Towards this end, we first characterized nectary anatomy and nectar volume. Second, we conducted a detailed transcriptomic analysis of nectaries from pre-anthetic, anthetic, and post-anthetic (post-fertilization) flowers to document gene expression patterns during nectary development and assess possible convergences in underlying genetic pathways. Finally, we conducted functional studies on key genes to test their direct role and putative interactions in nectary development and nectar production.

## 2 Materials and methods

### 2.1 Plant growth conditions

Inbred lines of *C. violacea* were grown from lab seed stock. A voucher was deposited in the vascular plant herbarium at the University of Alberta (ALTA; Hall & Bolton s.n., 20 February 2008; #813 from Hortus Botanicus, Amsterdam). Seeds were sown individually in 7.5 cm diameter pots containing sterilized (20 min, liquid, 121.1°C) Sun Gro Sunshine Mix (Agawam, Massachusetts, USA). All plants were grown in a growth chamber at the University of Alberta, Department of Biological Sciences with 16 h of full spectrum LED light at 22°C and 8 h of darkness at 18°C.

### 2.2 Histology and scanning electron microscopy (SEM)

Inflorescence tips, small buds (<1 mm wide), medium buds (1-1.5 mm wide), large buds (2.5-3 mm wide), flowers, and post-anthesis flowers were collected and fixed in FAA solution (50% EtOH, 5% glacial acetic acid, 10% formalin, 35% MilliQ water) and vacuum infiltrated as outlined previously (Hall et al., 2006; Patchell et al., 2011). Plant samples were then dehydrated in an ethanol series (50% to 100%). All ethanol solutions were kept at 4°C and samples were incubated for 2 hours. The 100% ethanol solutions were left overnight. Samples were infiltrated with CitriSolv (Decon Labs, USA) by changing to a 1:1 ethanol:CitriSolv solution, then changing to 100% CitriSolv. Each CitriSolv solution was incubated for two hours at room temperature with shaking. Samples were infiltrated with Tissue-Prep paraplast (Leica Biosystems, Canada) with 2-3 changes daily for five days then embedded in paraplast. Samples were sectioned to 8 μm using a Microm HM 325 (GMI, Inc., Ramsey, MN, USA) microtome prior to mounting. Slides were cleared with CitriSolv and dehydrated in isopropanol before staining with 0.025% Alcian blue and 0.01% Safranin O in 0.1M acetate buffer for two hours. Slides were examined using a Nikon (Tokyo, Japan) Eclipse 801 microscope with a Nikon DS-Ri1 photo system.

Samples used for scanning electron microscopy (SEM) were fixed and dehydrated as indicated above. After dehydration, samples were critical point dried with carbon dioxide using a CPD 030 critical point dryer (Bal-Tec AG, Liechtenstein, Germany). Specimens were then dissected and mounted on scanning electron microscopy stubs with conductive carbon tabs and sputter coated with gold using a Hummer 6.2 sputter coater (Anatech USA, Sparks, Nevada, USA). Finally, specimens were imaged using a ZEISS EVO 10 scanning electron microscope (Carl Zeiss AG, Oberkochen, Germany). Contrast and brightness of micrographs were adjusted using GIMP version 2.10.18 (https://gimp.org).

### 2.3 Nectar volume

Nectar volume of *C. violacea* was measured by pooling nectar from all the open flowers on each plant (2-7 flowers) in a capillary tube (Morrant et al., 2009). This measurement was taken for 20 plants and repeated at the same time each day for four consecutive days. Only flowers with visible nectar were measured. Individual flowers typically senesce three days after anthesis and stop producing nectar. Average nectar volume was calculated for each day and then graphed. A student’s t-test was run for binary comparisons between day 1-4.

### 2.4 RNA isolation and cDNA library preparation

Nectaries were collected from *C. violacea* flowers at three stages of development: pre-anthetic (buds 2.5-3 mm wide), anthetic (first day of anthesis) and post-anthetic (fertilized flowers with fruits at approximately 10 mm in length). RNA from these three developmental stages of four biological replicates were extracted to provide 1) an overview of gene expression at the end of nectary development and 2) insight into how gene expression changes before, at, and after anthesis. Nectary tissue was excised, flash frozen in liquid nitrogen, and stored at -80°C. Following: Carey et al., (2019), RNA was extracted from manually-ground frozen tissue using a Qiagen RNeasy micro kit (Hilden, Germany) and cDNA was generated using the Illumina TruSeq stranded mRNA LT sample prep kit RS-122-2101 (California, U.S.). In this case, mRNA for each sample was isolated using nucleomag beads (Macherey-Nagel, Düren, Germany). Samples were sent to The Center for Applied Genetics (TCAG) at the Toronto Sick Kids Hospital in Ontario, Canada where they were normalized, pooled and sequenced on a HiSeq 2500.

### 2.5 *De novo* transcript assembly, differential expression, and annotation

Raw reads were downloaded from the TCAG webserver and processed as in (Carey et al., 2019) using updated software (**Table S1**). The raw reads are available at the Sequence Read Archive (SRA) database (BioProject: PRJNA912718). After differential expression analysis with edgeR (Robinson et al., 2009), transcripts were classified as significantly differentially expressed if they had a log2 (fold-change) greater than four and a False Discovery Rate (FDR)-corrected p-value (α) less than 0.001. The ‘analyze_diff_expr.pl’ script, provided with Trinity (Haas et al., 2013), was used to generate a matrix of all significantly differentially expressed contig clustered transcripts, which was then used to generate a z-score heatmap in R (R Core Team, 2013).

We performed an additional z-score analysis with trinity transcripts filtered using TransDecoder.LongOrfs and TransDecoder.Predict to remove potential misassemblies. In total, 81,151 of 143,919 transcripts remained. A list of the 81,151 transcripts was used to extract significant transcripts from the original matrix file produced by ‘analyze_diff_expr.pl’, as well as from a list of all transcripts with expression greater than 100 TPM. Additionally, any transcripts with one or more biological groupings below 10 TPM, or with a coefficient of variation greater than or equal to 50, were removed.

All transcripts from the larger (original) trinity fasta file were annotated using BLASTx (Altschul et al., 1990) with default parameters and a local copy of the Araport11 protein database. Transcripts with the highest bit-score from the TAIR database were used as representative transcripts. Gene specific heatmaps were generated using ggplot2 and ggplot in R (R Core Team, 2013), respectively. Assembly completeness was determined using Benchmarking Universal Single Copy Orthologs (BUSCO) (Simao et al., 2015), and an ExN90 profile.

Transcripts unique to each stage were uploaded to the KEGG automatic annotation server (KAAS) using the bi-directional best hit against the following organism databases: *Arabidopsis thaliana* (Brassicaceae), *Brassica napa* (Brassicaceae), and *Tarenaya hassleriana* (Cleomaceae). Transcripts were considered unique if their expression was ≥ 10 TPM with a coefficient of variation < 50. A list of all KEGG entries was compiled, excluding most human diseases and other mammalian-exclusive categories. Of note, some categories were kept because they are convergent with pathways in plants and/or yeast.

### 2.6 Virus-induced gene silencing (VIGS)

Viral vector constructs were designed following (Carey et al., 2021). Tobacco rattle virus vectors pTRV1 (donor stock no. YL192) and pTRV2 (donor stock no. YL156) were obtained from The *Arabidopsis* Information Resource (TAIR; https://www.arabidopsis.org) using their stock center (*Arabidopsis* Biological Resource Center [ABRC], Ohio State University, Columbus, Ohio, USA; https://abrc.osu.edu). The pTRV2 vector is used for downregulating genes of interest (Ratcliff et al., 2001) and the pTRV1 vector assists with viral movement (Ziegler-Graff et al., 1991). Six new endogenous constructs were generated for this study using *C*. *violacea* mRNA: pTRV2-*CvANS*, pTRV2-*CvAG*, pTRV2-*CvAG*-*CvSHP*, pTRV2-*CvCRC*-*CvANS*, pTRV2-*CvSWEET9*-*CvANS*, and pTRV2-DN802_c0_g1_i4-*CvANS*. The *CvANS* construct was used as a marker gene and positive control. Treatment with pTRV2-*CvSHP* was explored in a preliminary round of VIGS but produced no remarkable floral phenotype and, as such, was abandoned in future trials.

All constructs were generated as follows. All cDNA was synthesized following manufacturer instructions using SuperScript III Reverse Transcriptase (Invitrogen), poly(T) primers, and random hexamer primers. All primers were designed using the transcriptomic data from this study. All amplification was done using Invitrogen recombinant Taq DNA Polymerase (Waltham, Massachusetts, USA) using the manufacturer protocol, 50 μL reaction volumes, and 35 cycles. All amplicons were verified using agarose gel electrophoresis, and colonies were screened *via* PCR with primers, 156F and 156R that span the TRV2 multiple cloning site (Gould and Kramer, 2007). Manufacturer protocols were used for each step unless otherwise noted. First, a 533 bp insert of *CvANS* was amplified using forward and reverse primers with added BAMHI [G^GATCC] and XHOI [C^TCGAG] restriction sites, respectively. Amplicons were purified using a QIAquick PCR purification kit and digested alongside empty TRV2 vector with NEB BAMHI and XHOI restriction enzymes (Ipswich, Massachusetts, USA). Digests were purified using the Quantum Prep Freeze ‘N Squeeze DNA Gel Extraction protocol with 200 μL pipette tips and 2 mL tubes in lieu of spin columns. Eluate was further purified using ethanol precipitation (https://projects.iq.harvard.edu/hlalab/resources-0). Digests were ligated together using NEB T4 DNA ligase and immediately transformed using One Shot™ TOP10 Chemically Competent *E*. *coli*. *Escherichia coli* was incubated for 24 h at 37°C in Miller LB broth (Sigma-Aldrich, Burlington, Massachusetts, USA) containing 50 μg/mL kanamycin. The pTRV2-*CvANS* construct was verified using colony PCR, and colonies containing the appropriately sized plasmids were extracted using a GeneJET Plasmid Miniprep Kit (Thermo Fisher, Waltham, Massachusetts, USA), verified using agarose gel electrophoresis, and transformed into chemically competent Agrobacterium GV3101; cells were prepared and transformed according to protocol (Luo et al., 2008). All media used to grow *Agrobacterium* contained 50 μg/mL kanamycin, 50 μg/mL gentamycin, and 25 μg/mL rifampicin. Plasmids from transformed Agrobacterium were verified *via* restriction digestion and agarose gel electrophoresis, and finally sanger sequencing. Agrobacterium containing the appropriate pTRV2-*CvANS* vector were grown for 48 h at 28°C and mixed 1:1 with sterile 50% glycerol prior to storage at -80°C.

All other vectors were made following the same protocol. Amplicons from *CvCRC*, *CvSWEET9*, and DN802_c0_g1_i4 were ligated to pTRV2-*CvANS* vectors using XBAI [T^CTAGA] and BAMHI restriction sites; *ANS* acting both as a positive control and marker gene for facilitated phenotyping. The pTRV2-*CvAG* construct was generated using BAMHI and XHOI restriction sites, as with pTRV2-*CvANS*. The *CvSHP* amplicon was then ligated to the pTRV2-*CvAG* vector using XBAI and BAMHI restriction sites. No *CvANS* marker was used for the *CvAG* or *CvAG-CvSHP* constructs because downregulation of *CvAG* is distinct. Viral constructs were verified for off‐target silencing using siFi21 (Lück et al., 2019).

*Agrobacterium tumefaciens* was prepared for DNA transformation as previously described (Carey et al., 2021). All vectors were transformed into *A*. *tumefaciens* using calcium chloride heat‐shock transformation. For each, 100 ng of purified construct was combined with 250 µL of competent *A. tumefaciens*. Transformants were plated on LB media containing the aforementioned antibiotics. Transformants were then screened as before using 156F and 156R primers, and glycerol stocks were made and stored at −80°C (1:1 ratio of 50% glycerol and overnight *A. tumefaciens* culture).

The vacuum infiltration protocol, which has been shown to be an effective infiltration method with *C. violacea*, was modified from Carey et al. (2021). For each vector, *A*. *tumefaciens* cultures were serially inoculated up to 1000 mL cultures containing antibiotics, 1mM MES buffer and 0.02 mM acetosynringone. A 1:1 ratio of pTRV1 cultures were also serially inoculated up to 1000 mL. The final cultures were grown until they reached an OD600 between 0.8-1, and then immediately centrifuged and resuspended in infiltration buffer (10 mM MES, 10 mM MgCL2 and 0.2 mM acetosyringone) to an OD600 of 4.0 ± 0.1 and left for four hours to acclimatize. *Agrobacterium* containing pTRV1 should be inoculated 1-2 hours prior to pTRV2 cultures because they have a slower growth rate. An OD600 of four was chosen because it has been reported to achieve greater yields, and when pTRV2 and pTRV1 are combined their OD600 values half to an optimal OD600 of 2.0 (Wang et al., 2006). The serial inoculation was halted at OD600 0.8 to capture log-phase growth. The pTRV2 and pTRV1 suspensions were combined prior to infiltration at a 1:1 ratio. Silwet L-77 surfactant was added to each mixture at 100 μL/L. Groups of seedlings were extracted from the soil, rinsed in reverse osmosis water, briefly air-dried, submerged in *Agrobacterium*, and placed in a vacuum chamber. The chamber was evacuated to -20 inHg and held for 2 minutes. Vacuum pressure was then quickly released, and plants were rinsed and planted in fresh soil. Finally, plants were grown at 22°C for 16 h and 18°C for 8 h because it was found that lower temperatures consistently resulted in better VIGS efficacy in *Petunia* (Broderick and Jones, 2014).

All treated plants began showing phenotypes five weeks post-inoculation, and phenotypes lasted until plant senescence. Phenotypes for construct pTRV2-*CvANS* were scored based on reduction in maroon pigmentation in petals, which is hereafter referred to as yellowing, i.e., a reduction in anthocyanins resulted in petals with increased yellow pigmentation. A flower was scored as having a moderate phenotype when at least two petals displayed obvious yellowing. Flowers were scored as having a strong phenotype if all four petals displayed obvious yellowing. Flowers with less yellowing than moderate flowers, but which were distinct from untreated flowers, were scored as having a mild phenotype. There were no observed instances of only a single petal yellowing.

The yellowing phenotype assisted with scoring of *CvCRC*, *CvSWEET9*, and DN802_c0_g1_i4 constructs. Phenotypes for pTRV2-*CvCRC*-*CvANS* were scored based on complete or partial absence of nectary. Yellowed flowers with complete nectaries, and non-yellow flowers without nectaries were also recorded because it is possible for only a single gene to be silenced even with multiple gene constructs. Phenotypes for pTRV2-*CvSWEET9-CvANS* were scored based on visual inspection of nectary gland for presence of nectary droplets using a dissection microscope. Phenotypes for pTRV2- DN802_c0_g1_i4-*CvANS* were indistinguishable from pTRV2-*CvANS*.

Phenotypes for *pTRV2-CvAG* and *pTRV2-CvAG-CvSHP* were scored based on *AG* silenced phenotypes in *Arabidopsis* because of conservation of ABC gene function (Mizukami and Ma 1997). Silencing efficacy was based on the extent of repetition of perianth whorls and the absence of reproductive whorls. For both constructs, presence/absence of nectaries, absence of reproductive whorls, and repetition of perianth whorls were noted. Plant tissue from treated and control plants was excised, flash-frozen in liquid nitrogen, and stored at -80°C. Phenotypes were imaged using a Nikon SMZ 1500 dissecting microscope (Nikon, Tokyo, Japan) and a handheld digital Canon DS126181 camera (Canon, Tokyo, Japan). Images were standardized, scaled, color balanced, and assembled into figures using Inkscape version 0.92.5 (https://inkscape.org) and GIMP.

## 3 Results

### 3.1 Morphology and nectar production in *Cleome violacea*

**Figure 2:**
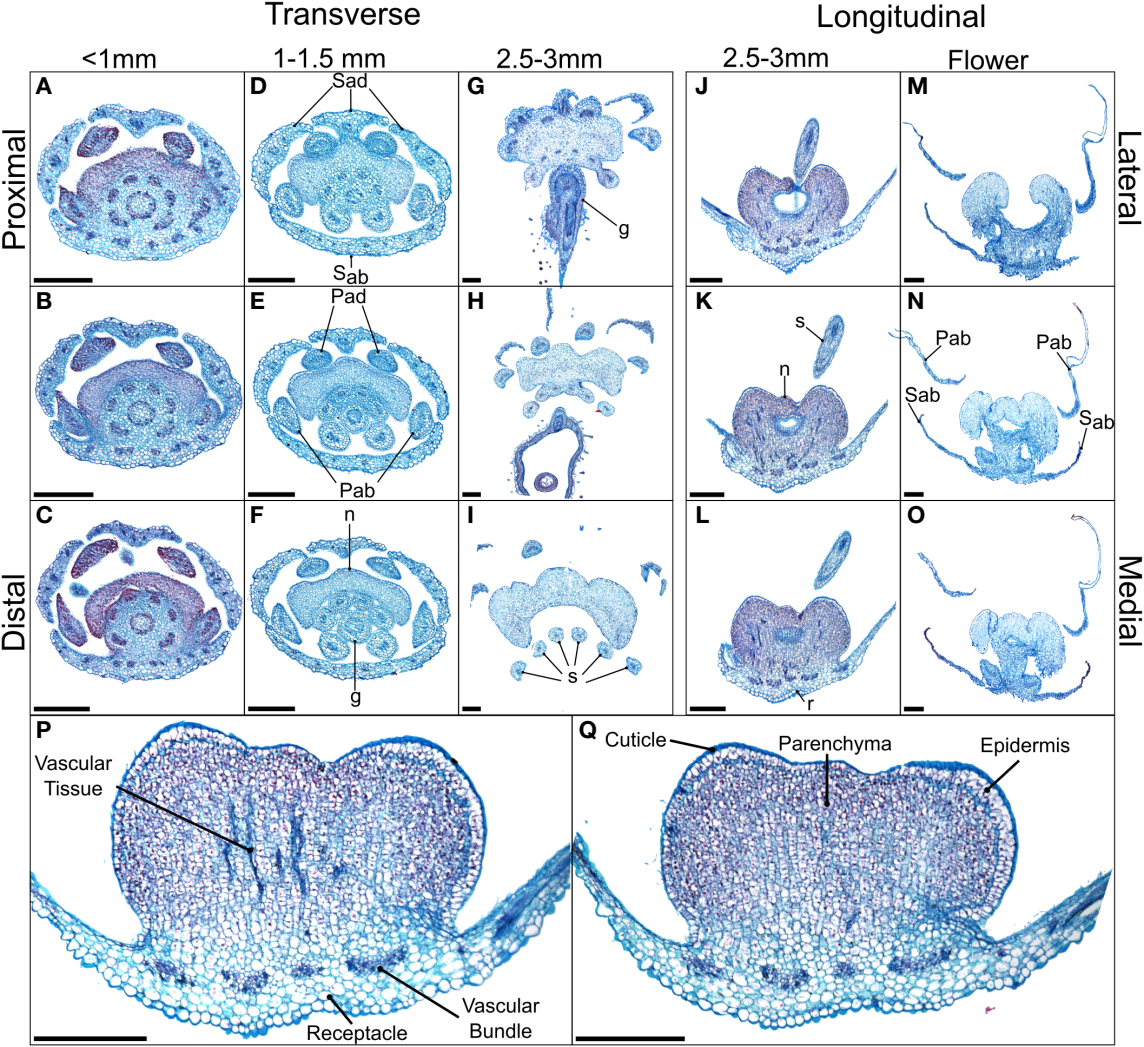
Alcian blue/safranin O-stained sections of *Cleome violacea* nectaries at pre-anthetic, anthetic and post-anthetic stages. From left to right: **(A–C)** small, **(D–F)** medium, and **(G–I)** large buds in transverse view with proximal-distal indicating relative distance to receptacle. **(J–L)** Large bud and **(M–O)** flowers in longitudinal view with lateral-medial indicating relative distance from center. **(P, Q)** Longitudinal view of 20 μm slices of the same large floral bud with and without vascular tissue, respectively. Scale bars = 250 μm. Sad = adaxial sepal; Sab = abaxial sepal; Pad = adaxial petal; Pab = abaxial petal; s = stamen; g = gynoecium, r = receptacle.

**Figure 3:**
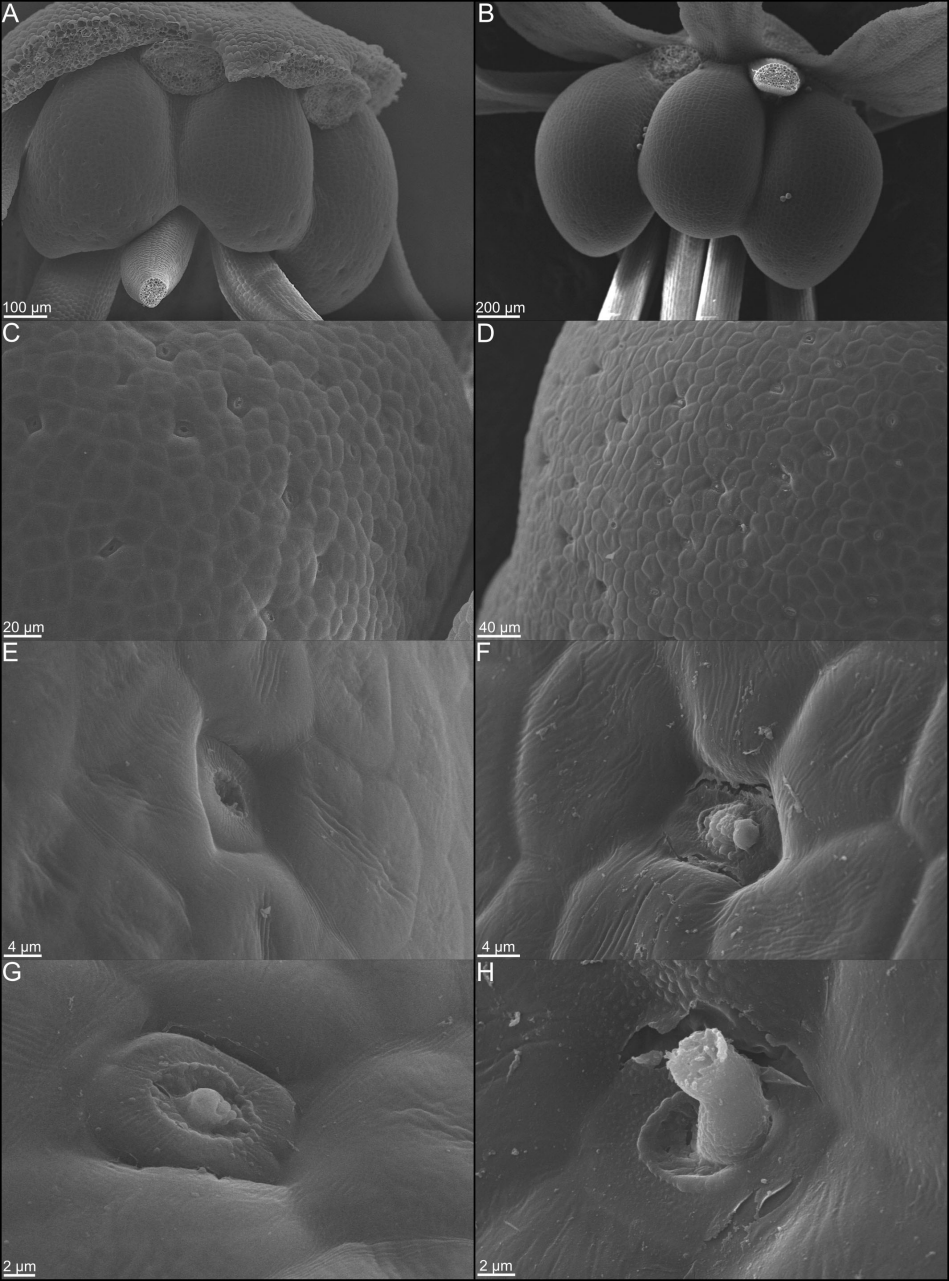
Scanning electron micrographs of whole nectaries from Cleome violacea at **(A)** pre-anthetic and **(B)** anthetic stages. **(C)** Distribution of nectarostomata on pre-anthetic nectary lobe and **(D)** anthetic nectary lobe. Examples of nectarostomata from **(E–G)** bud and **(F–H)** anthetic flowers.

Anthetic nectaries of *C. violacea* are adaxially positioned between petals and stamen. These nectaries are prominent due to their relatively large size (i.e., roughly half the size of an abaxial petal). Nectaries are tri-lobulate with two larger lateral lobes and a smaller central lobe at anthesis (**Figures 1B–E**). Following the terminology of Nepi (2007), these structured nectaries have prominent epidermis, and mostly consist of specialized parenchyma with vascular tissue interspersed throughout (**Figure 2**). Nectar is secreted *via* nectarostomata, which are present prior to anthesis (**Figure 3**). Floral nectaries are first visible late in development when developing stamens reach the same length as petals. At this stage, sepals and petals are growing, stamens have differentiated into filaments and anthers, and the gynoecium is formed with papillate stigma. Nectaries mature concordantly with stamens and reach maturity just prior to anthesis (**Figure 1B**). Nectary primordia are visible in small buds (**Figure 2**) when sepals are maturing.

**Figure 4:**
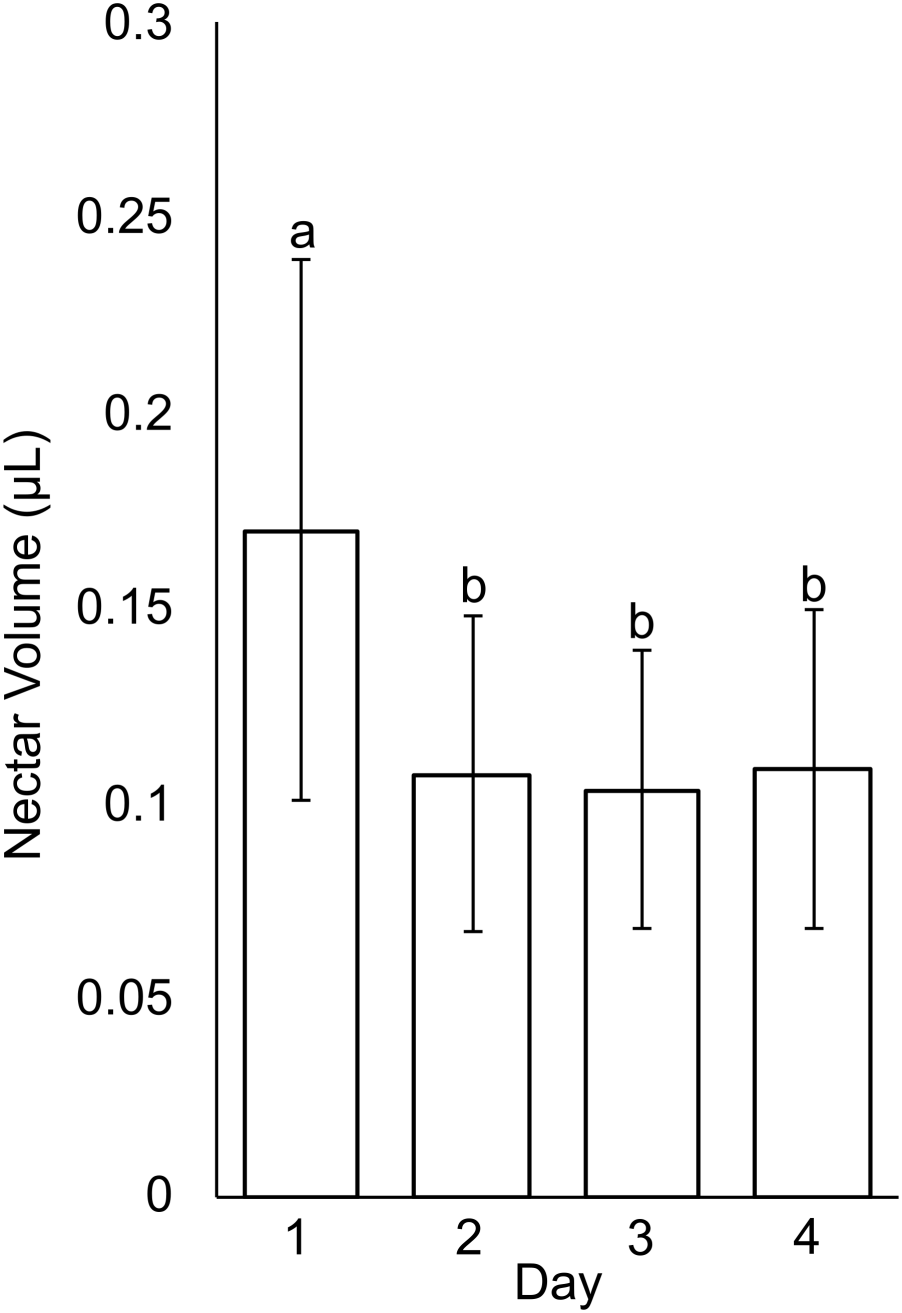
Nectar volume from *Cleome violacea* flowers taken on first day of anthesis (Day 1) and three days post-anthesis (Days 2, 3, and 4). Averaged value of flowers from 20 plants. Significance measured using paired, one-tailed, student’s t-tests (α < 0.01).

In small buds (<1.0 mm wide), nectaries are oblong and marginally lobed. At this stage, cells appear parenchymal with no differentiation of epidermis or vascular tissue, although the cuticle is apparent (**Figures 2A–C**). Medium buds (1-1.5 mm wide) have more pronounced lobes with differentiated epidermis (**Figures 2D–F**). Large buds (2.5-3.0 mm wide) have larger lobes comprised of parenchymal cells which make up the bulk of the nectary (**Figures 2G–I**). Epidermal cells are 1-2 layers thick on medial and lateral nectary lobes (**Figure 2Q**). In large buds, vascular tissue is distributed throughout the specialized parenchyma and likely connects with other vasculature near the receptacle base (**Figure 2P**) and with nectarostomata on the nectary surface (**Figure 3**). These nectarostomata are present on large buds (**Figure 3A**) and anthetic flowers (**Figure 3B**). Nectaries produce a low volume of nectar that decreases in volume after anthesis (**Figure 4**). Anthetic nectaries produce an average of 0.17 ± 0.07 μL) of nectar (**Figure 4**). Nectar volume decreases after day 1 but remains stable over three consecutive days of sampling at ~ 0.11 ± 0.04 μL) (**Figure 4**).

### 3.2 Expression profiles show distinct gene expression patterns pre to post anthesis

The transcriptome is of suitable quality and completeness for downstream analyses. The transcriptomic read depth averaged 19.9 million reads across 12 biological replicates, totaling just over 239M paired end trimmed reads. Median Phred scores are between 34 and 39 for each base pair of all 143,919 Trinity transcripts (**Table S2**), which indicates a base call accuracy between 99.7% and 99.99% (data not shown). The E90N50 value of the assembled transcriptome is 2227, and peaks at 2259 for Ex93 and Ex94 (**Figure S1**). A peak around Ex90 generally indicates a high level of transcriptome completeness. Further, the Benchmarking Universal Single Copy Orthologues (BUSCO) analysis of Viridiplantae orthologues (Simao et al., 2015) revealed that the transcriptome was 99.6% complete with 2 fragmented BUSCOs (**Table S3**).

**Figure 5:**
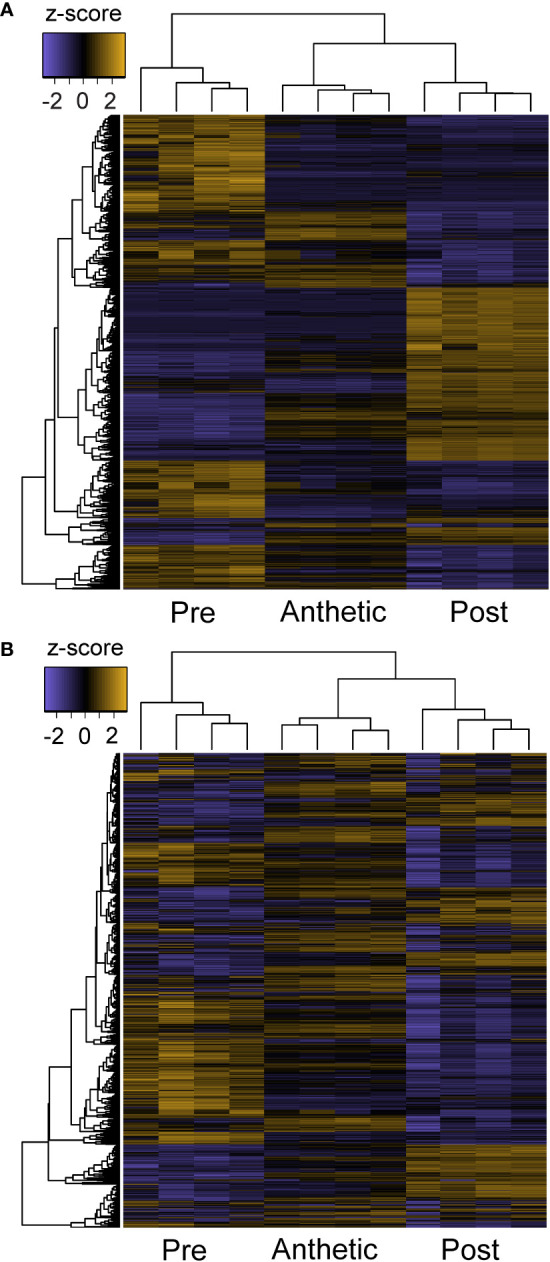
Z-score heatmaps of **(A)** all differentially expressed transcripts and **(B)** transcripts with TPM > 100 from *Cleome violacea* pre-anthetic, anthetic, and post-anthetic nectaries.

Heatmap patterns of gene expression of pre-anthetic, anthetic and post-anthetic nectaries are consistent across two distinct thresholds. We compared all 4521 significantly differentially expressed transcripts, as well as the 1214 transcripts above 100 TPM (with a coefficient of variation < 50 in one or more biological groupings) (**Figure 5**). Pre and post-anthetic nectaries have opposing expression profiles, such that transcripts upregulated in pre-anthetic nectaries are generally downregulated in post-anthetic nectaries. Anthetic nectaries have no large clusters of up or downregulated transcripts and appear to be partially transitional, although they have a few unique clusters of differential expression (**Figure 5**). Expression patterns of transcripts filtered using TransDecoder were similar to the unfiltered list for significantly differentially expressed transcripts, as well as those above 100 TPM (**Figure S5**). In sum, each of the three developmental stages is genetically distinct.

To provide additional insight into gene regulatory networks governing nectar secretion and nectary development, we also assembled the highest expressed transcripts across all stages from the TransDecoder-filtered dataset. This list included 20 of the highest expressed transcripts from pre-anthetic, anthetic and post-anthetic stages that were significantly differentially expressed in pairwise comparisons, and the top 20 highest overall expressed transcripts that were not differentially expressed. Due to overlap, there were 56 transcripts in total (40 among the differentially expressed and 16 non-differentially highly expressed transcripts). A few transcripts matched to the same genes leaving 51 unique accessions. Out of the 51, eight had no obvious role specific to nectary function (e.g., ubiquitous cellular process; **Table S4**). Five were related to photosynthesis, 14 to water transport and sugar production, 16 to stress response and six to cell growth. *YABBY5*, which can dimerize with *CRC* (Gross et al., 2018) was also among the highest expressed (**Table S4**). Putative gene function was estimated using gene description information from TAIR (www.arabidopsis.org), and a brief review of the literature.

### 3.3 Energy metabolism and hormonal regulation across nectary development

Genetic networks were assessed to determine which categories were active at each sampled stage of nectary development. KEGG analyses revealed several categories that had different relative counts (i.e., putative orthologs) in one or more stages: energy metabolism, biosynthesis of secondary metabolites, translation, replication and repair, environmental adaptation, and cell growth and death. We interpret greater counts as greater biological activity. A difference of three or less was disregarded to account for any potential noise in the data, e.g., invalid isoforms created during the assembly process. In pre-anthetic nectaries, oxidative phosphorylation (35 relative to 25 in the other two stages), and thermogenesis (40 relative to 32 in the other two stages) are the only subcategories with a greater number of putative orthologs (**Table S5**). Mitochondrial oxidative phosphorylation in plants is known to provide ATP for cellular functions (e.g., sucrose metabolism) and is tightly linked to photosynthesis (Braun, 2020). However, photosynthetic processes are similar between all stages (**Table S5**). Anthetic nectaries have no categories with greater hits than the other two stages (**Table S5**). Post-anthetic nectaries have increased biological activity in two categories, replication and repair, and cell growth and death; three of the five subcategories for cell growth and death are directly related to yeast (**Table S5**), i.e., nectary yeast are likely contributing to ortholog abundance in this category. Overall, most categories have a similar number of putative orthologues across all stages.

**Figure 6:**
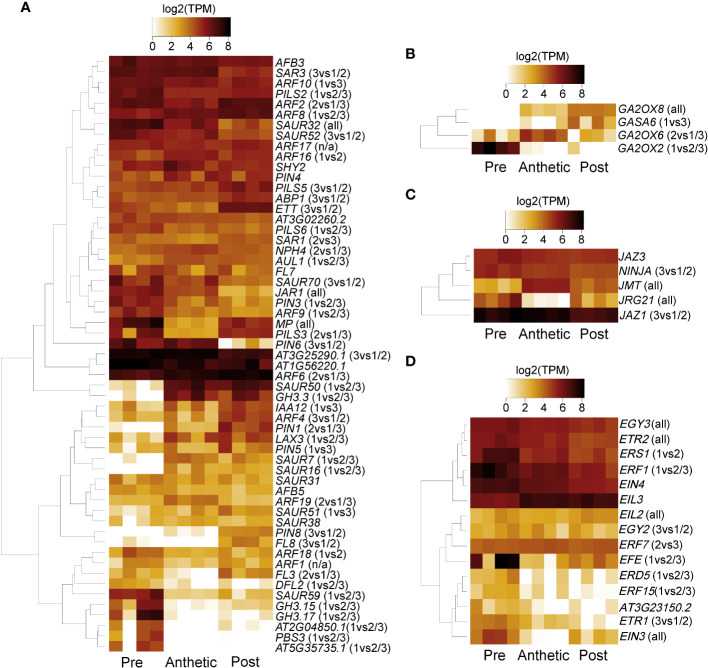
A heatmap of phytohormone-related transcripts expressed in pre-anthetic, anthetic, and post-anthetic nectaries of *Cleome violacea* displayed in log2(TPM). Representative transcripts of genes related to **(A)** auxin, **(B)** gibberellic acid **(C)** jasmonic acid, and **(D)** ethylene. Significance displayed in brackets. 1 = pre-anthetic; 2 = anthetic; 3 = post-anthetic.

Although plant hormone signalling is important in nectary development and nectar secretion, there was no indication of differences between hormone signalling related orthologs between stages, based on the KEGG analysis (**Table S5**). However, expression analyses indicated significant differential expression in genes related to these pathways (**Figure 6**). Auxin, JA, and GA are known to regulate transcriptional expression related to nectar secretion (Slavkovic et al., 2021), and ethylene interacts synergistically with auxin (Muday et al., 2012), although to our knowledge has no direct link to nectaries. Examples of highly expressed transcripts were *AUXIN RESPONSE FACTOR 6* (*ARF6*) and *JASMONATE ZIM-DOMAIN PROTEIN 1* (*JAZ1*) (**Figures 6A, C**). Three *GIBBERELLIN 2-OXIDASE* genes were expressed in pre-anthetic, anthetic and post-anthetic nectaries, respectively (**Figure 6B**). We also found significant upregulation of multiple ethylene related transcripts in pre-anthetic nectaries (e.g., *ETHYLENE RESPONSE FACTOR 1* (*ERF1*) and *ETHYLENE FORMING ENZYME* (*EFE1*). (**Figure 6D**). These data suggest that a combination of auxin, JA, and GA influence nectary development and nectar secretion in *C. violacea.*

### 3.4 Yeast and bacteria are present on Cleome *violacea* nectaries

**Figure 7:**
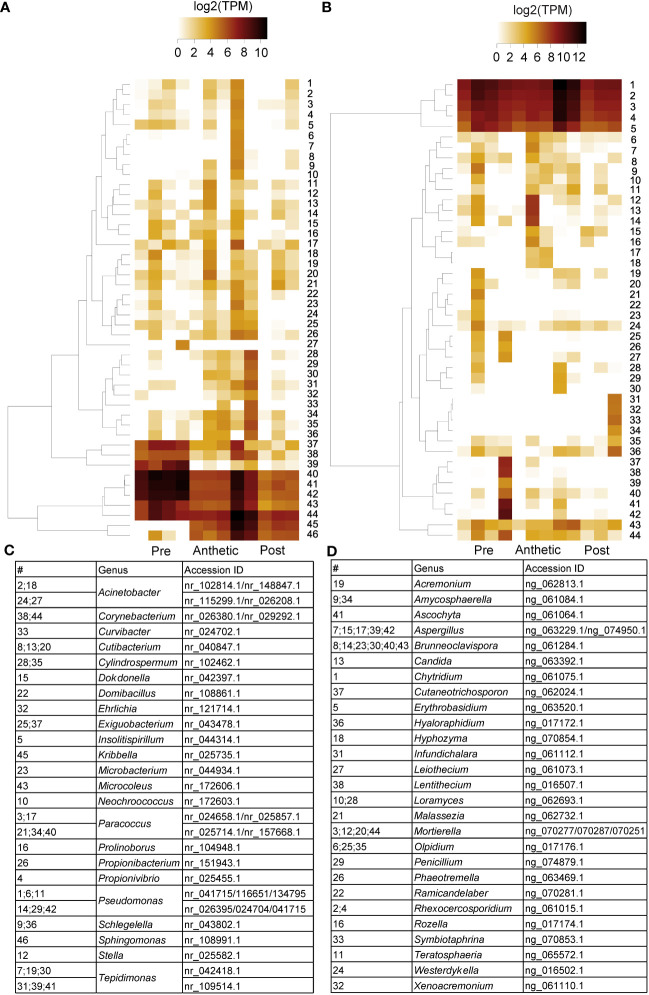
Heatmap of **(A)** 16s bacterial rRNA and **(B)** 18s fungal rRNA related transcripts in pre-anthetic, anthetic, and post-anthetic nectaries of *Cleome violacea* displayed in log2(TPM). Genera and NCBI accessions of respective transcripts for bacteria and fungi outlined in **(C, D)**, respectively.

Transcriptomic and SEM data provide evidence that yeast and bacteria colonize C*. violacea* nectaries. There are a total of 46 and 44 hits (e-value < 1e-50) to bacterial and yeast-related rRNA in the *C. violacea* transcriptome, respectively (**Figure 7**). Ribosomal rRNA can still be present in poly(A)-enriched libraries in appreciable percentages (Kim et al., 2019), which is valuable for finding non-plant related expression. Generally, expression of fungal and bacterial rRNA was inconsistent across biological replicates and stages; suggesting that colonization may be replicate specific. However, there are a few instances where expression is consistent across replicates and stages, which may indicate an established biological interaction (**Figure 7**). These data are further supported by the obstructions surrounding and within the nectarostomata (e.g., what appears to be budding yeast cells) (**Figures 3F–H**) and are consistent with the abundance of yeast related KEGG terms (**Table S5**). For example, there are nearly twofold more KEGG terms related to the yeast cell cycle in post-anthetic nectaries (43) than pre-anthetic (22) or anthetic (20) nectaries (**Table S5**). Carotenoid related genes *FLAVONOL SYNTHASE* 1 (*FLS1*), *PHYTOENE SYNTHASE (PSY*), and *CHALCONE SYNTHASE* (*CHS*) are also highly expressed at various developmental stages (**Table 1** and **Figure S2**). All three genes have purported roles in combating biotic stress (Dao et al., 2011; Havaux, 2014; Naparlo et al., 2019).

**Table 1:** Genes of interest not directly implicated in nectary development with relative expression values from our transcriptomic dataset, putative roles, and relevant citations.

Gene	SDE	Putative Role	Citation
*TPL*	≈	Co-repressor of *AG* which is recruited by *AP2* in floral organ identity	Krogan et al., 2012
*STM*	↓S2 vs S1/S3	Controls carpel development and requires the function of *AG*	Scofield et al., 2007
*SEP3*	↓S2 vs S1/S3	Functions in combination with B and C class genes to activate *CRC*	Lee et al., 2005a
*FTM4*	↑S3 vs S2/S3	Encodes an intracellular LRR protein that interacts with *AG*	Torti et al., 2012
*SEP4*	≈	Functions in combination with B and C class genes to activate *CRC*	Lee et al., 2005a
*SOC1*	≈	Functions together with *FUL* to promote development of inflorescence	Preston et al., 2011
*AP1*	↑S3 vs S1/S2	Regulates fatty acid biosynthesis with *CRC* in *Arabidopsis*	Han et al., 2012
*GI*	≈	Regulates miR172, which in turn regulates *BEN* and *ROB* in *Petunia*	Jung et al., 2007
*FLD*-*LIKE*	≈	Required for systemic acquired resistance in *Arabidopsis*	Singh et al., 2013
*FUL*	↓S3 vs S1/S2	Expressed in *Arabidopsis* nectaries from stage 9 to stage 14	Baum et al., 2001
*CO*	↑S1 vs S2/S3	Promotes *SOC1* and *FT*	Jung et al., 2007
*SEP1*	≈	Functions in combination with B and C class genes to activate CRC	Lee et al., 2005a
*YABBY5*	↑S2 vs S1/S3	Can dimerize with *CRC via* the *YABBY* domain	Gross et al., 2018
*TPS*	↑S3 vs S1/S2	Levels of trehalose change in parallel with sucrose; regulates stomatal conductance and water use.	Lunn et al., 2014
*FLS1*	↑↑S3 vs ↑S2 vs S1	Accumulation of flavanols may increase survival of yeast by reducing oxidative stress	Naparlo et al., 2019
*PSY*	↑S2 vs S1/S3	Carotenoid oxidation products function as a plant stress signal	Havaux, 2014
*CHS*	↑S3 vs S1/S2	Linked to resistance of biotic stress	Dao et al., 2011

SDE, Significant Differential Expression with arrows representing either up or down regulation of expression between developmental stages (≈ indicates no SDE). S1, pre-anthetic nectary; S2, anthetic nectary; S3, post-anthetic nectary.

### 3.5 Dynamic expression patterns of genes involved in nectar and nectary formation

After establishing global patterns and active biological networks, we examined expression patterns of 17 genes of interest with uncertain roles, nine genes known to be involved in nectar production, and ten genes with direct roles in nectary formation (e.g., expression in *Arabidopsis* nectaries) (**Tables 1**–**3**; **Figure S2**). These analyses reveal dynamic expression patterns from pre to post-anthetic nectaries. Seven genes linked to nectar production are significantly upregulated in either pre-anthetic and/or anthetic nectaries: *BAM1, PIN6, MYB21, SWEET9, JAZ, G20X* and *JMT* (**Table 2**). This pattern mirrors the onset of nectar production. Interestingly, expression profiles for nectary development genes (**Table 3**) are generally opposite to nectar production (**Table 2**). That is, of the genes examined with established roles in nectary development, most transcripts are downregulated in pre-anthetic nectaries, and three of ten genes explored are evenly expressed across all stages (**Table 3**). Downregulated genes include *PI*, *AG*, *ARF6*, *ARF8*, and *STY* (**Table 3**). Of the 17 genes with uncertain roles, six are evenly expressed across all three developmental stages: *SEP1/4, TOPLESS* (*TPL*)*, SUPRESSOR OF OVEREXPRESSION OF CO 1* (*SOC1*)*, GIGANTEA* (*GI*), and *FLOWERING LOCUS D LIKE* (*FLD-like*). Of the remaining, no clear pattern emerges (**Table 1**).

**Table 2:** Genes of interest with direct roles in nectar production with relative expression values from our transcriptomic dataset, putative roles, and relevant citations.

Gene	SDE	Putative Role	Citation
*CWINV4*	≈	Hydrolyzes sucrose into fructose and glucose; knockouts do not produce nectar	Ruhlmann et al., 2010
*SBE2.2*	≈	Involved in starch synthesis; upregulated early in development in ornamental tobacco	Ren et al., 2007
*BAM1*	↑S1 vs S2/S3	Starch breakdown; upregulated during secretory stage in Cucurbita pepo	Solhaug et al., 2019
*PIN6*	↓S3 vs S1/S2	Expression level is positively correlated with nectar production in *Arabidopsis*	Bender et al., 2013
*MYB21*	↑S2 vs S1/S3	Induces negative feedback loop on jasmonate biosynthesis	Reeves et al., 2012
*SWEET9*	↓S3 vs S1/S2	Required for nectar secretion in *Arabidopsis*	Lin et al., 2014
*JAZ*	↓S3 vs S1/S2	Represses jasmonic acid (JA) signalling in a negative feedback loop	Chico et al., 2008
*GA2ox6*	↑S2 vs S1/S3	Inactivates gibberellic acid (GA), which increases expression of genes involved in nectar production	Wiesen et al., 2016
*JMT*	↑S2 vs S1/S3	Forms Methyl Jasmonate from JA; JA conjugates are linked to increased nectar production.	Radhika et al., 2010

SDE, Significant Differential Expression with arrows representing either up or down regulation of expression between developmental stages (≈ indicates no SDE). S1, pre-anthetic nectary; S2, anthetic nectary; S3, post-anthetic nectary.

**Table 3:** Genes of interest with direct roles in nectary formation with relative expression values from our transcriptomic dataset, putative roles and relevant citations.

Gene	SDE	Putative Role	Citation
*AP3*	↑S3 vs S1/S2	Downregulation disrupts nectary placement and nectar secretion in *Arabidopsis*	Baum et al., 2001
*PI*	↓S1 vs S3	Downregulation disrupts nectary placement and nectar secretion in *Arabidopsis*	Baum et al., 2001
*AP2*	≈	Downregulation disrupts nectar secretion in *Arabidopsis*	Baum et al., 2001
*AG*	↓S1 vs S2/S3	Redundantly activates *CRC* with *SHP1/2*	Morel et al., 2018
*ARF8*	↓S1 vs S2/S3	Affects nectary size and gene expression redundantly with *AUXIN RESPONSE FACTOR* 6 (*ARF6*) in *Arabidopsis*	Reeves et al., 2012
*ARF6*	↓S1 vs S2/S3	Affects nectary size and gene expression redundantly with *ARF8* in *Arabidopsis*	Reeves et al., 2012
*BOP2*	≈	Promotes the formation of nectary glands independent of *CRC*	Mckim et al., 2008
*SHP1*/2	↑S2 vs S3	Redundantly regulates *CRC* with *AG*	Morel et al., 2018
*STY*	↓S1 vs S2/S3	Controls nectary development in *Aquilegia* independent of CRC	Min et al., 2019
*CRC*	≈	Essential but not sufficient for nectary formation in the core eudicots.	Lee et al., 2005b

SDE, Significant Differential Expression with arrows representing either up or down regulation of expression between developmental stages (≈ indicates no SDE). S1, pre-anthetic nectary; S2, anthetic nectary; S3, post-anthetic nectary.

### 3.6 Functional studies demonstrate key roles of *CvAG, CvSHP, CvCRC*, and *CvSWEET9* in nectary development and nectar secretion

VIGS experiments tested the putative function of five genes. Four of the five genes targeted for downregulation were highly expressed and have established roles in nectary development and nectar production: *CvAG*, *CvSHP*, *CvCRC*, and *CvSWEET9* (**Tables 2**, **3** and **Figure S2**). We also downregulated an uncharacterized transcript (DN802_c0_g1_i4) because it was among the highest expressed in the transcriptome, has a similar profile to *CvSWEET9*, and has no significant match to either the TAIR11 database or the nr database, despite an ORF of 375 bp (**Figure S3**). It partially matches AT412520.1 (e-value = 8.19e-4) from the TAIR11 database and MW419336 (e-value = 6.33e-7) from the nr database. These hits were not considered further because of their large e-values. 

**Figure 8:**
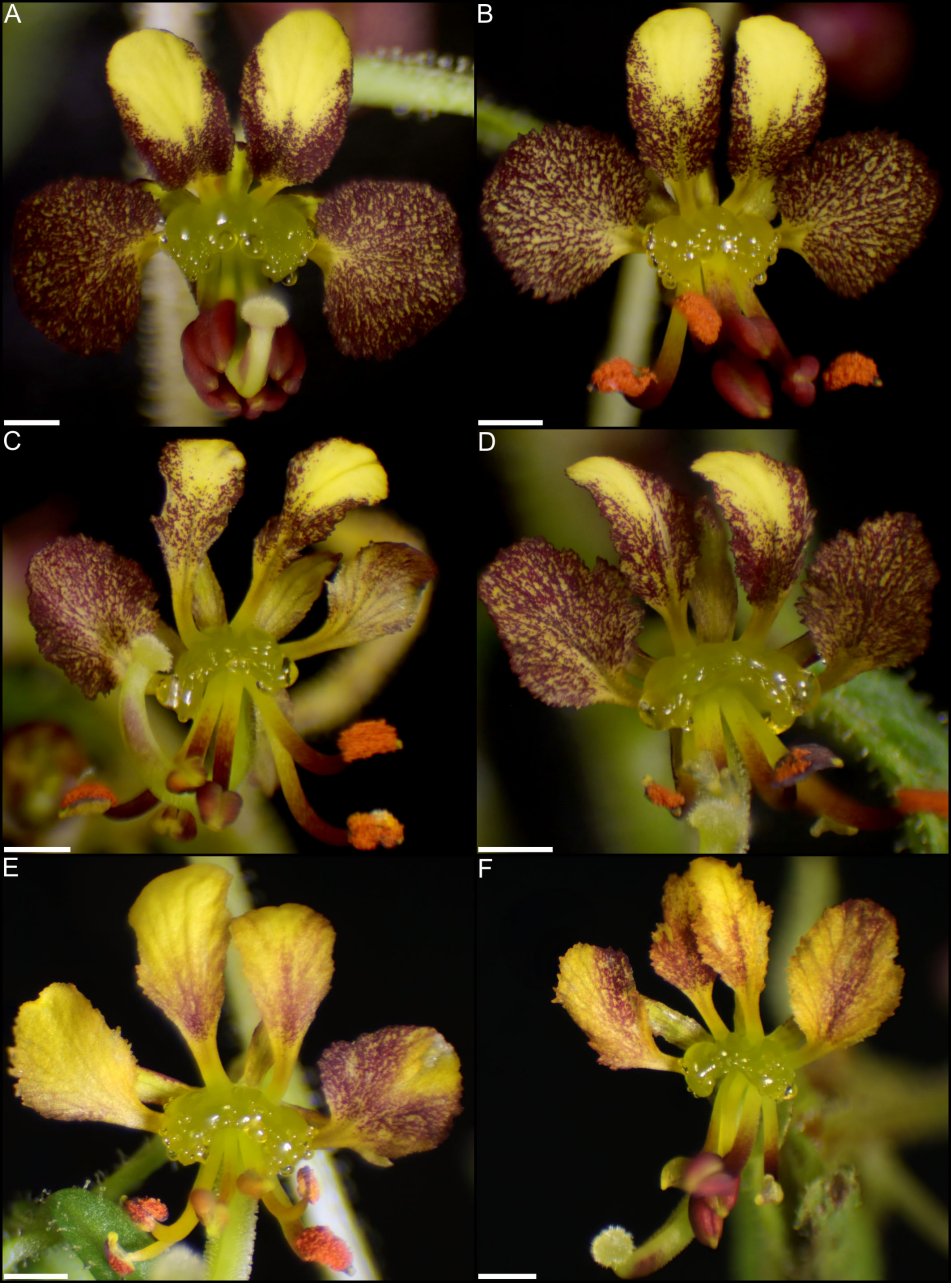
*Cleome violacea* flowers from untreated and treatment control groups. **(A)** Untreated newly anthetic flower and **(B)** maturing flower. pTRV2-MCS treated flower displaying **(C)** moderate and **(D)** mild viral phenotype. **(E, F)** pTRV2-*CvANS* treated flowers displaying moderate yellowing petal phenotypes. Scale bars = 1 mm.

*ANS* was used both as a positive control vector (pTRV2-*CvANS*) and as a marker gene to facilitate scoring of phenotypes for *CvCRC* (pTRV2-*CvCRC-CvANS*), *CvSWEET9* (pTRV2-*CvSWEET9-CvANS*), and DN802_c0_g1_i4 (pTRV2-DN802*-CvANS*). Untreated *C. violacea* and plants treated with pTRV2-MCS constructs were also used as controls (**Figures 8A–D**). Treatment with pTRV2-*CvANS* produced flowers with primarily yellow pigmentation on adaxial and abaxial petals (**Figures 8E, F**). Yellowing is purported to be from a disruption of the anthocyanin production pathway and was the visual marker used for other constructs because it is not expected to alter the form or function of nectaries. *CvANS* is only moderately expressed in post-anthetic nectaries (**Figure S2**).

With exception of pTRV2-DN802_c0_g1_i4 (**Figure S4**), all other treatment groups had marked phenotypes related to nectary and nectar formation. DN802_c0_g1_i4 was highly expressed in pre-anthetic and anthetic nectaries, and relatively downregulated in post-anthetic nectaries (**Figure S2**). Treatment with pTRV2-DN802_c0_g1_i4*-CvANS* resulted in flowers that were phenotypically indistinguishable from the *CvANS* control (**Figure S4**; **Figures 8E, F**), despite a high efficacy and mortality among treated flowers relative to control (**Table S6**). It is plausible that this transcript is related to water and/or nutrient transport because of its unusually high mortality during silencing (**Table S6**), absence of discernible silencing phenotype (**Figure S4**), and similar expression profile to *CvSWEET9* (**Figure S2**), but further research is required. 

**Figure 9:**
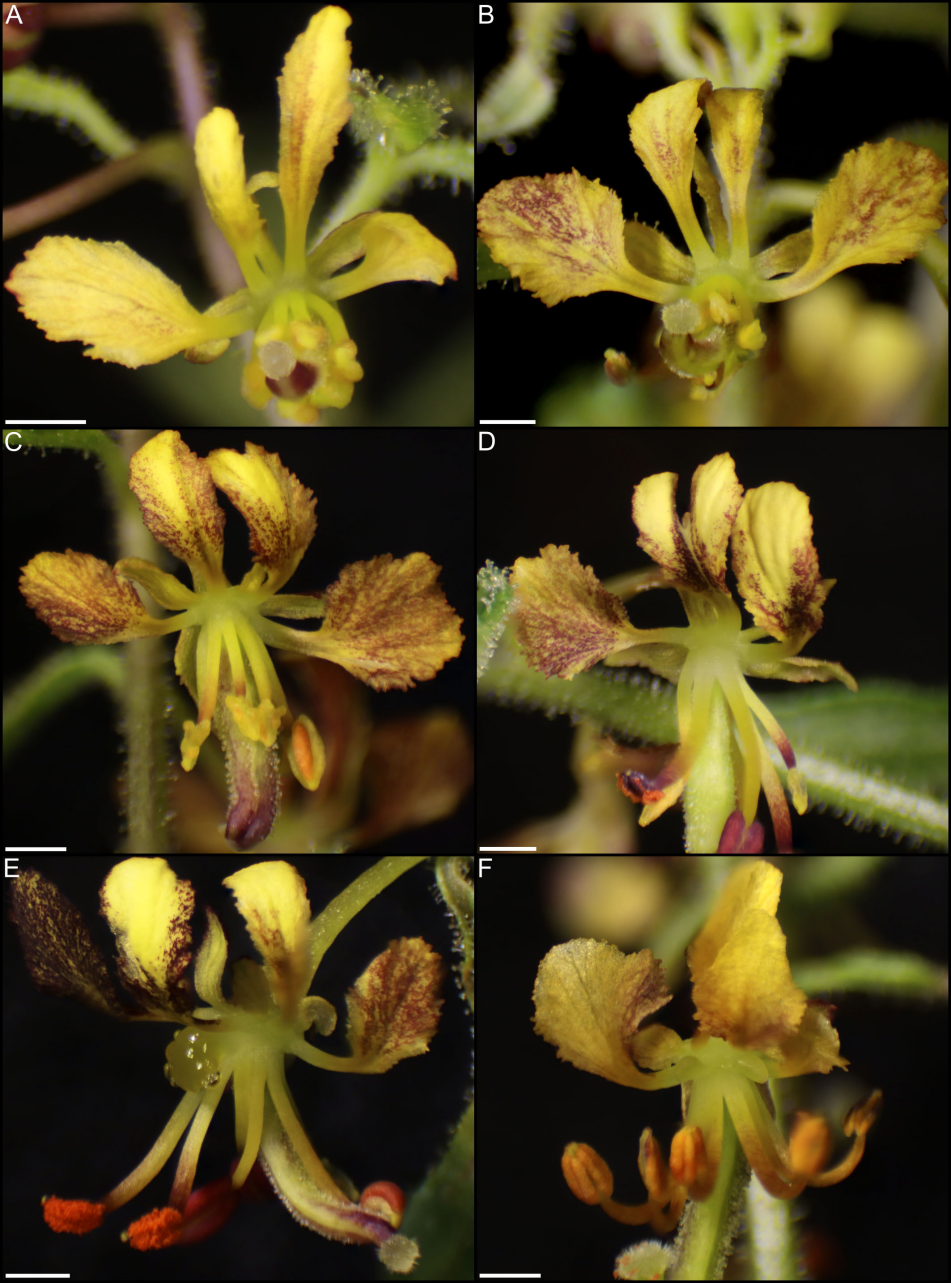
Flowers of *Cleome violacea* treated with pTRV2-*CvCRC-CvANS* constructs. **(A)** Flower with strong yellowing phenotype and no nectary. **(B)** Flower with moderate yellowing phenotype and no nectary. **(C)** Flower with moderate yellowing phenotype, no nectary, and enlarged gynoecium. **(D)** Flower with moderate yellowing phenotype and no nectary. **(E)** Flower with half normal and half yellowing petals with partially absent nectary. **(F)** Flower with strong yellowing phenotype and reduced lateral nectary lobes. Scale bars = 1 mm.

**Figure 10:**
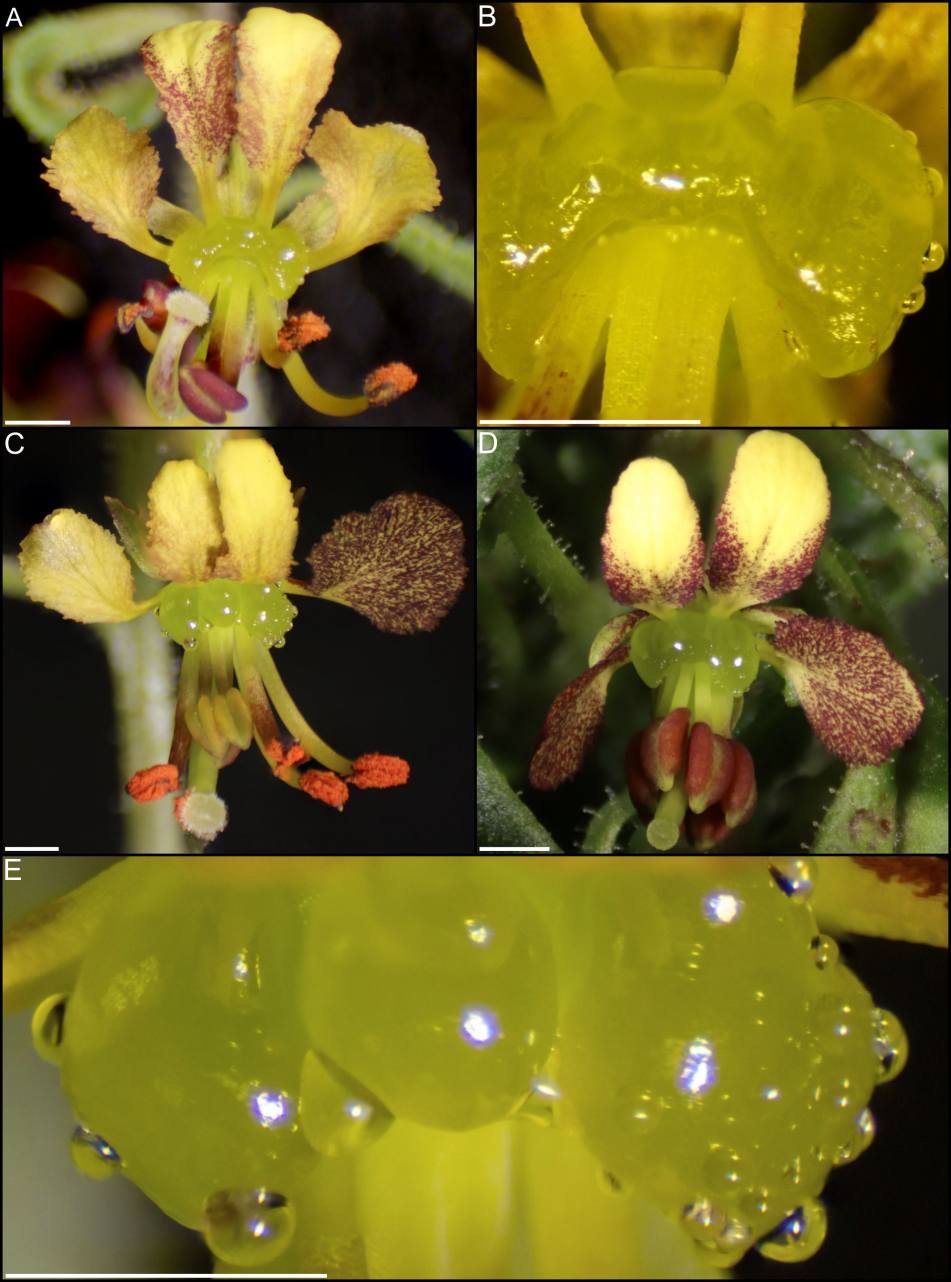
Flowers of *Cleome violacea* treated with pTRV2-*CvSWEET9-CvANS* constructs. **(A)** Flower with moderate yellowing and nectary with reduced nectar accumulation. **(B)** Magnified view of nectary in A. **(C)** Flower with partial yellowing and partial normal phenotype. **(D)** Flower with near-normal pigmentation and reduced nectar production. **(E)** Magnified nectary from C displaying decreased nectar accumulation correlating with yellowing phenotype. Scale bars = 1 mm.

Functional studies suggest the role of *CvCRC* and *CvSWEET9* in nectary and nectar formation, respectively. *CvCRC* is expressed across all three developmental stages investigated, but with no significant difference between stages (**Table 3**). In contrast, *CvSWEET9* was downregulated in post-anthetic nectaries as compared to pre-anthetic and anthetic nectaries (**Table 2**). Treatment with pTRV2-*CvCRC-CvANS* resulted in either partial or total loss of nectaries in all flowers with yellowing phenotype (**Figure 9**; **Table S6**). Treatment with pTRV2-*CvSWEET9-CvANS* produced flowers with a visible reduction in nectar on their nectaries (**Figure 10**), and no detectable sugar using a refractometer (n = 10; data not shown).

**Figure 11:**
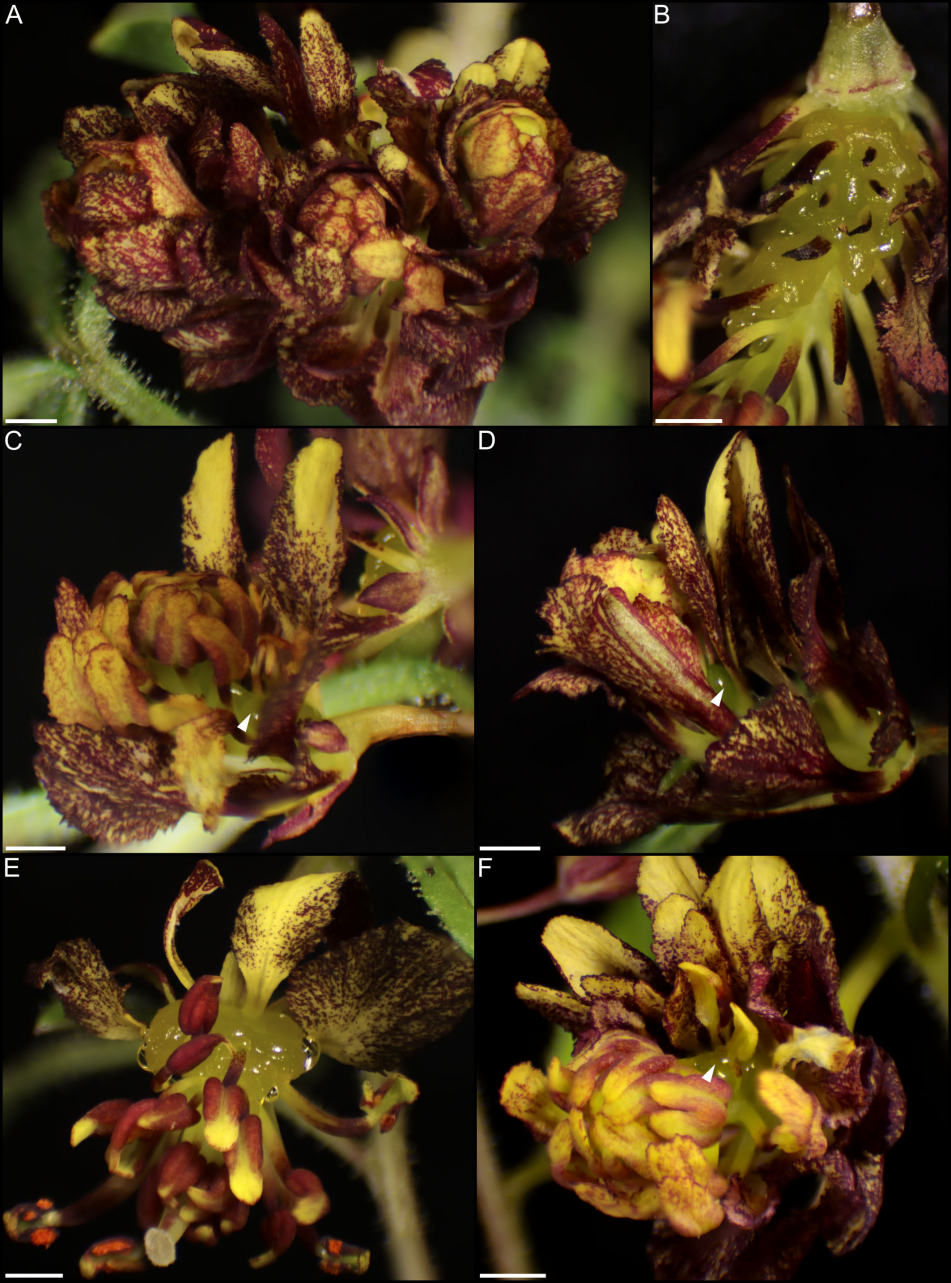
Flowers of *Cleome violacea* treated with pTRV2-*CvAG* constructs. **(A)** Flower with repeating perianth whorls. **(B)** Nectary from flower similar to A with petals removed. **(C)** Flower with normal adaxial petals, repeating perianth whorls and adaxial nectary. **(D)** Flower with repeating perianth whorls and distally positioned nectary. **(E)** Flower with petaloid stamens and adaxial nectary. **(F)** Flower with repeating perianth whorls and adaxial nectary. White arrowheads indicate nectary position. Scale bars = 1 mm.

**Figure 12:**
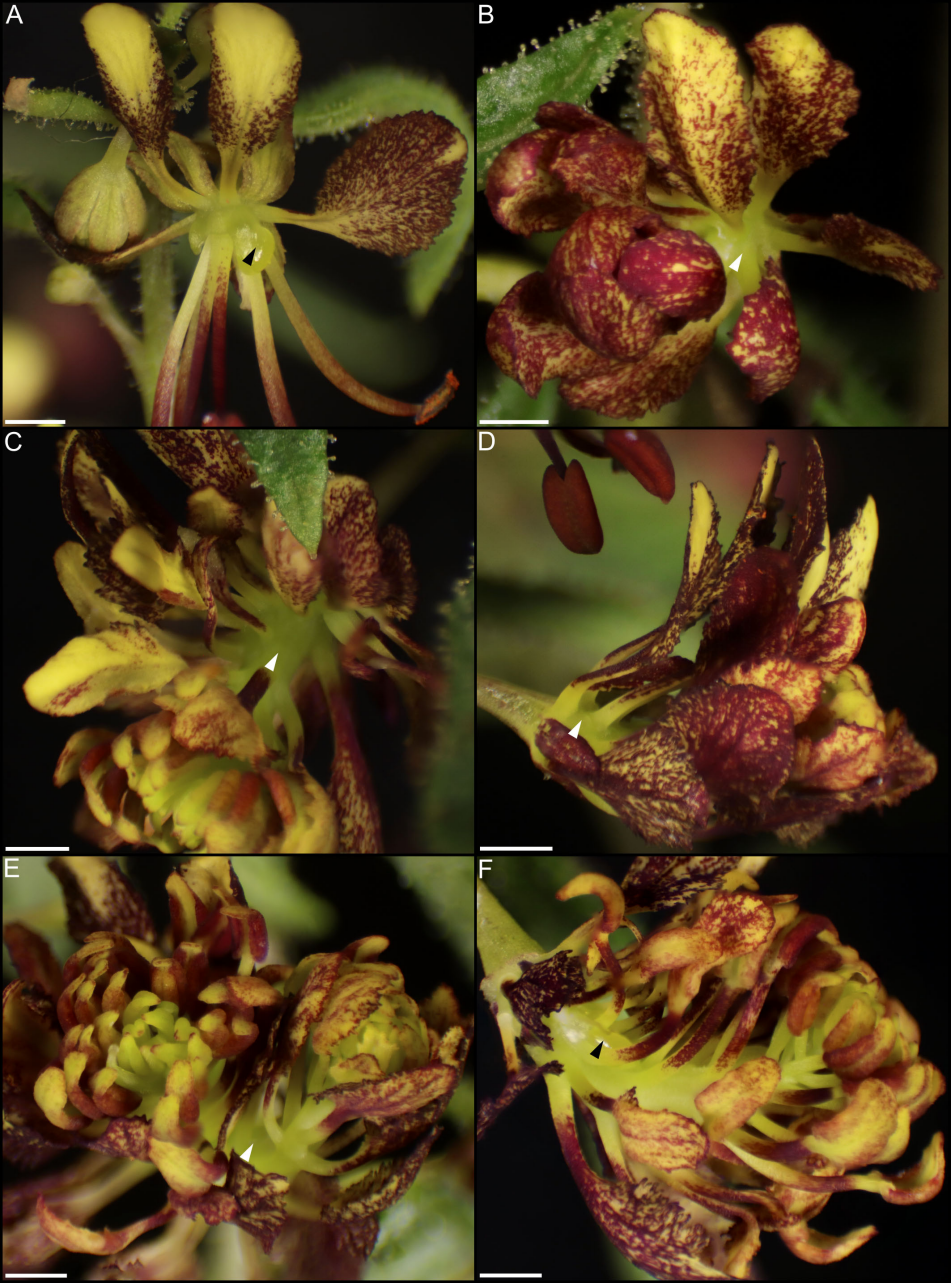
Flowers of *Cleome violacea* treated with pTRV2-*CvAG-CvSHP* constructs. **(A)** Flower with partial nectary. **(B–E)** Flowers with repeating perianth whorls and no nectary. **(F)** Flower with repeating perianth whorls and partial nectary. Black and white arrowheads represent reduced and absent nectary, respectively. Scale bars = 1 mm.

Individual and combined constructs of *CvAG* and *CvSHP* demonstrate these genes are functionally redundant in regulation of nectary formation. Treatment with pTRV2-*CvAG* and pTRV2-*CvAG-CvSHP* produced flowers without reproductive whorls and repeating perianth (**Figures 11**, **12**). Flowers treated with pTRV2-*CvAG* still produced nectaries, but their position and structure were altered relative to untreated flowers (**Figure 11**). This misplacement is likely due to a loss of reproductive whorls and repeated morphology. Flowers treated with pTRV2-*CvAG-CvSHP* generally produced no nectaries, although occasionally they were present and reduced in size (**Figures 12A, F**).

## 4 Discussion

### 4.1 *Cleome violacea* have structured nectaries that produce nectar secreted *via* nectarostomata

The nectary of *C. violacea* is striking in that it is a prominent feature of the flower, due to its large size and location (**Figure 1**). The nectary is adaxially positioned between stamens and adaxial petals, contributing to monosymmetry of the flower in addition to petal color patterning and reproductive organ curvature. Nectaries appear late in development, well after initiation of stamens and gynoecium. Once formed, mature nectaries are a large 3-lobulate structure (**Figures 1**–**3**). *Cleome violacea* nectaries are characteristic of many other structured nectaries (Nepi, 2007): (1) the nectary epidermis has thick cuticle, (2) the nectary parenchyma is made up of small, dense cells, and (3) the vasculature is interspersed throughout the nectary and likely connects with vascular bundles in the receptacle (**Figure 2P**). Presence of nectarostomata on nectaries of *C. violacea* has been described previously (Erbar and Leins, 1997b). In the annular nectary of *Cleomella sparsifolia* (= *Cleome sparsifolia*), nectarostomata appear on abaxial tips (Lee et al., 2005b), suggesting nectarostomata may be common in Cleomaceae. Nectarostomata are modified stomata that secrete carbohydrate rich solutions for pollinator reward; a similar genetic pathway regulates both nectarostomata and unmodified stomata (Pillitteri et al., 2008; Baylis et al., 2013).

Nectar is known to be secreted *via* a few different methods, and most commonly *via* nectarostomata in a granulocrine or eccrine based manner (Nepi, 2007). Outlined by Roy et al. (2017) eccrine based secretion begins with the breakdown of starch and subsequent synthesis of sucrose, which is then transported out of the cell and hydrolyzed before secretion out of nectarostomata in droplets of nectar. Key to the export of sucrose is *SWEET9*, which is essential for sugar transport in nectaries of *Arabidopsis*, *Brassica rapa*, and *Nicotiana* (Lin et al., 2014). Another relevant gene, *CWINV4* is important for nectar formation in *Arabidopsis*, specifically cleaving sucrose in the extracellular space which has the effect of moving water towards sugar, forming nectary droplets (Ruhlmann et al., 2010). In *C. violacea*, multiple lines of evidence support eccrine-based nectar secretion. First, there are nectarostomata on the nectary surface (**Figures 3C–H**) which likely connect with the vasculature present throughout parenchymal tissue (**Figure 2P**). Second, both *CvSWEET9* and *CvCWINV4* are highly expressed in pre-anthetic and anthetic nectaries (**Table 2** and **Figure S2**). Additionally, we identified 14 highly expressed transcripts that are related to sugar production or water transport, e.g., five of which are related to aquaporins found in *Aquilegia* (Singh et al., 2020) (**Figure S5** and **Table S4**). Finally, nectar secretion is lessened when *CvSWEET9* is completely downregulated (**Figure 10**). In sum, nectary secretion in *C. violacea* is dependent on *CvSWEET9*, as demonstrated for *Arabidopsis*, *Brassica* and *Nicotiana*, which supports its key role in sucrose export across the core eudicots (Lin et al., 2014).

Nectar is secreted at anthesis, accumulates on C. *violacea* nectary lobes (**Figure 1E**) and has a low average secretion volume (0.17 ± 0.07 μL) (**Figure 4**). This volume is lower than averages of wild populations of other species of Cleomaceae: *Cleomella serrulata* (0.85 ± 0.96 μL) and *Polanisia dodecandra* (0.63 ± 0.32 μL) (Higuera-Diaz et al., 2015). However, it is similar to the average volume produced by one species of Brassicaceae: *Erysimum mediohispanicum* (0.136 ± 0.010 μL). The differences in nectar volume may be in part explained by flower size as *C*. *violacea* has much smaller flowers than *C. serrulata* and *P. dodecandra.* It may also reflect different pollinator environments; flowers of *C. serrulata* and *P. dodecandra* have a wide range of visitors and somewhat overlap in geography in some areas of North America (Higuera-Diaz et al., 2015). It is also unclear if there is a reduction of nectar in lab-grown inbred lines of *C. violacea* relative to wild populations. No empirical pollination study has been conducted on *C. violacea* to date, which is native to Spain (GBIF.org), so there is no information on which pollinators would be attracted to and rewarded by its nectar.

### 4.2 Cv*CRC, CvSHP*, and *CvAG*, exhibit conserved roles with other core eudicots in nectary formation

*CRC* is essential for nectary formation in *Arabidopsis*, *Petunia*, *Pisum*, and *Medicago* in addition to having an important role in carpel formation (Baum et al., 2001; Lee et al., 2005b; Fourquin et al., 2014). As with these taxa and other core eudicots (Lee et al., 2005b; Slavkovic et al., 2021), *CvCRC* is expressed in developing nectaries without any significant difference in gene expression patterns from late-stage buds to post-anthetic flowers (**Table 3** and **Figure S2**). Like with other species, loss of *CvCRC* resulted in an absence of nectaries (**Figure 9**), which demonstrates that *CvCRC* is essential for nectary formation in *C. violacea*. While strong *CsCRC* expression in nectaries of *C. sparsifolia* implied the conserved role of *CRC* (Lee et al., 2005b), this study provides the first functional evidence of the direct contribution of *CRC* to nectary formation in Cleomaceae. Since the nectaries of *C. sparsifolia* are annular, forming a ring around the stamen base, as compared to the adaxial position of *C. violacea* nectaries (**Figure 1**), these data indicate that upstream regulators of *CRC* are likely important for nectary position and morphology within Cleomaceae flowers. In *Medicago* and *Pisum*, inconspicuous nectaries form at the base of the staminal tube. Like with Fabaceae, nectaries of *Arabidopsis* are found at the base of stamens, although in this instance forming six glands on the abaxial side. Nectaries in *Petunia* form a ring at the base of the gynoecium (Morel et al., 2018). Interestingly, unlike knockout or knockdowns of *CRC* in *Arabidopsis* (Alvarez and Smyth, 1999; Alvarez and Smyth, 2002), Fabaceae (Ferrandiz and Fourquin, 2014), and poppy (Orashakova et al., 2009), we did not observe many notable changes to gynoecium or fruit formation in *CvCRC* knockdowns (but see **Figure 9C**). Additional studies are necessary to explore the extent of *CRC’s* role in gynoecium development and its conservation in *C. violacea*. The highly expressed *YABBY5* (**Table 1**, **Figure S2**) should also be explored due to its ability to dimerize with *CRC* (Gross et al., 2018), i.e., it may share a role with CRC in *C. violacea*. *CRC* homologs have variable importance in carpel formation across core eudicots (Morel et al., 2018), which also warrants further examination in *C. violacea*. As shown in *Arabidopsis* (Pinyopich et al., 2003), we predict a high redundancy of gene function for gynoecial formation in *C. violacea* given its importance to plant fitness.

MADS-box genes *AG* and *SHP* act redundantly upstream of *CRC* in both *Arabidopsis* and *Petunia* to initiate nectary development (Morel et al., 2018). The regulatory roles of these genes appear to be conserved in nectary formation of *C. violacea.* Both *CvAG* and *CvSHP* are strongly expressed across all stages of development (**Table 3** and **Figure S2**). Treatment with pTRV2-*CvSHP* or pTRV2-*CvAG* alone is insufficient to prevent nectaries from forming (**Figure 11** & **Table S6**). Treatment with pTRV2-*CvSHP* alone has no effect on floral phenotype (**Table S6**) but treatment with pTRV2-*CvAG* disrupts the formation of whorls 3 and 4 (**Figure 11**). These phenotypes in *C. violacea* are like *Arabidopsis ag-1* mutants (Baum et al., 2001). Only doubly silenced flowers do not produce nectaries, although they are otherwise like flowers treated with pTRV2-*CvAG* (**Figure 12**). Our data is consistent with the model from Wollmann et al. (2010) which shows a balance between *AP2* and *AG* activities, i.e., in pTRV2-*CvAG* treated flowers, stamens occasionally appear petaloid (**Figure 11F**). Thus, there is the possibility that the overlapping of whorls may be the condition which contributes to nectary formation because all the ABC genes are expressed in nectaries to some degree (**Table 3** and **Figure S2**). When flowers are treated with pTRV2-*CvAG*, and petals form haphazardly, nectary tissue surrounds each petal at the base of the flower and the lobe-like structure is lost. Perhaps this is because nectary tissue here has no boundary due to the absence of reproductive whorl (**Figure 11B**). These results are consistent with those observed in *Arabidopsis* and *Petunia* in that *CRC* expression is dependent on both *AG* and *SHP* lineages (Morel et al., 2018). It is striking that the upstream regulators are likely shared between these three taxa. However, like Morel et al. (2018) our data cannot distinguish whether this shared regulation is due to a single evolutionary origin of nectaries or due to the conservation of *CRC* in carpel development.

Intriguingly, when *CvTCP1* is downregulated in *C. violacea*, nectaries are altered with phenotypes ranging from reduced lobes to complete absence (Carey et al., in prep). Like *CRC*, the regulatory pathway upstream of *TCP1* is unclear, although the key contribution of *TCP* homologs towards many types of floral monosymmetry has been demonstrated across angiosperms (Preston and Hileman, 2009; Hileman, 2014b; Wessinger and Hileman, 2020). Given that expression domains of *AG* and *SHP* are much broader across the flowers, other genetic factors are required for restriction of nectaries to a single whorl (Morel et al., 2018). In *C. violacea*, *CvTCP1* may be involved, at least indirectly. As noted above, functional data for floral nectaries to date has been conducted on flowers whose nectaries are distributed evenly around floral organs (e.g., circular around *Petunia* gynoecium and at the base of all stamens in *Arabidopsis*), unlike the adaxial positioning of the nectary in *C. violacea.* Thus, adaxial floral identity may be required for nectary formation in *C. violacea*, although it is unclear if *TCP1* has a direct role in nectary initiation. Functional studies of Cleomaceae with annular nectaries, such as *Tarenaya hassleriana*, would inform on decoupling nectary position and identity in the family.

Less is known about nectary size than initiation. In *Petunia*, *BEN* and *ROB* are important for nectary size (Morel et al., 2018), whereas *BOP1/2* impacts nectary size in *Arabidopsis* (Mckim et al., 2008). It is perhaps unsurprising that no *BEN* or *ROB* homologs were expressed in nectaries of *C. violacea*, but *CvBOP2* is expressed throughout all stages examined (**Table 3** and **Figure S2**). Unlike *BEN* and *ROB*, the interactions between *BOP1/2* and other floral homeotic genes, with regards to nectary formation and size, are not as well understood (Slavkovic et al., 2021). Further experiments are needed to determine whether *CvBOP2* contributes to nectary size in *C. violacea*. In our analysis of highly expressed transcripts (**Figure S5** and **Table S4**), six transcripts are potentially linked to cell growth in nectaries, although they have only been characterized in leaves (e.g., *EXL2*) and roots (e.g., *PRX44*) (Schröder et al., 2009; Marzol et al., 2022). Future studies should explore genes similar to those identified in this study, as well as earlier stages of nectary development. Altogether, these expression patterns suggest that pathways determining nectary size are not conserved across the core eudicots.

Gene expression data suggests additional conservation as well as deviation in the genetic pathway of nectary development between *Arabidopsis* and *C. violacea*. Transcriptomic data shows many genes important for nectary formation are conserved across *Arabidopsis* and *Cleome*, including ABC genes *AG, AP2, AP3, PI*, and *MADS-box gene SHP* (**Table 3** and **Figure S4.2**). Notably, *AqSTY* has been shown as essential for nectary formation in *Aquilegia* (Min et al., 2019) and *CvSTY* is expressed in nectaries of *C. violacea*. While expression is low, it is significantly differentially expressed and down regulated in pre-anthetic flowers (**Table 3** and **Figure S2**). This co-expression presents a tantalizing hypothesis that *CvSTY* and *CvCRC* are not mutually exclusive pathways in *C. violacea* nectary development. *STY* likely interacts with *CRC* in developing carpels of *Arabidopsis* (Kuusk et al., 2002) such that interactions in other floral structures are feasible. In addition, *STY* is also linked to auxin biosynthesis (Baylis et al., 2013), which is important to nectary development.

### 4.3 The nectar of *Cleome violacea* is complex, as is its secretion method

Nectar is a multifaceted sugar solution that changes in composition over time and includes microorganisms as well as secondary metabolites made by both plant and microbes (Alvarez-Perez et al., 2012; Chappell and Fukami, 2018; Parachnowitsch et al., 2019; Liao et al., 2021; Jacquemyn et al., 2021). Our data are consistent with bacteria and fungi colonization of *C. violacea* nectaries (**Figure 7**) and reflect complexities in these interactions. Unsurprisingly, many of the identified microorganisms from this study are commonplace in soil and/or have been previously isolated from nectar (e.g., *Sphingomonas*, *Pseudomonas*, and *Erythrobasidium*) (**Figure 7**) (Alvarez-Perez et al., 2012; Jacquemyn et al., 2013). However, the exact nature of the relationship (i.e., mutualism, commensalism, or parasitism) cannot be determined with gene expression data alone, especially since there was variation across replicates. Nonetheless, we found six transcripts that potentially play a role in combating biotic stress from our analysis of highly expressed transcripts (**Figure S5** and **Table S4**), e.g., *LIPID TRANSFER PROTEIN 2* (*LTP2*) and *β-GLUCOSIDASE 19* (*BGLU19*) (Molina and García-Olmedo, 1997; Li et al., 2019) KEGG counts also showed enriched plant-pathogen interactions (26, 25, and 26 in pre-anthetic, anthetic, and post-anthetic nectaries, respectively.) (**Table S5**). Further, compounds typically produced by nectar-associated microbial communities (e.g., alcohols, isoprenoids, and ketones) (Rering et al., 2018) are difficult to distinguish with transcriptomics because many of these metabolites are also produced by the plant. Additionally, yeasts are known to chemically alter metabolites already present in nectar (Vannette and Fukami, 2016).

In all stages of developing nectaries of *C. violacea*, we found roughly even KEGG counts of carotenoid and flavonoid biosynthesis (~14 and ~8 across stages, respectively) (**Table S5**). Flavonoids have antioxidant activity, which reduces reactive oxygen species (ROS) in *Arabidopsis* (Vannette and Fukami, 2016), and they have also been linked to the reduction of *E.coli* fimbria, which may reduce biofilm formation (Lee et al., 2011). *FLS1* is significantly upregulated in anthetic and post-anthetic nectaries (**Table 1** and **Figure S2**). Additionally, accumulation of flavanols have also been shown to increase survival of yeast by reducing oxidative stress (Naparlo et al., 2019). Thus, flavonoid accumulation may be a way to inhibit bacterial biofilms while simultaneously supporting symbiotic yeast. *CHS*, which is highly expressed in our transcriptome (**Table 1**, **S4** and **Figure S2**), is also linked to resistance against biotic and abiotic stress such as UV, temperature, wounding, and bacteria (Dao et al., 2011). However, even though their role in the reduction of ROS can potentially impact biotic stress, carotenoids are more commonly linked to abiotic stress (Havaux, 2014), pollinator attraction (Cazzonelli, 2011), and photoprotection (Demmig-Adams, 1990), so further research is required.

Phytohormone expression in *C. violacea* is complex with evidence supporting convergence to other eudicots. In Brassicales, phytohormones play an important role in gland development and nectar secretion (Slavkovic et al., 2021). In both *Aquilegia* and *Arabidopsis*, auxin is linked to nectary initiation *via ARF6* and *ARF8* (Nagpal et al., 2005; Reeves et al., 2012) to nectar production *via PIN6* (Bender et al., 2013). *Cleome violacea* nectaries express multiple *ARFs* and *PINs* across development (**Figure 6A**), although not all expression is identical to that in *Arabidopsis* (**Tables 2**, **3**). All PINs serve to promote the flow of auxin between cells (Křeček et al., 2009), so there is likely conservation of function between Cleomaceae and Brassicaceae. Auxin however does not function alone, and gland development is complicated by phytohormone interactions. For example, JA is positively and negatively regulated by auxin and GA, respectively. Both GA (Wiesen et al., 2016) and JA (Radhika et al., 2010) are linked to nectar secretion (Slavkovic et al., 2021), e.g., JA is positively correlated with nectar production. For other biological processes (e.g., seedling development) there is substantial crosstalk between auxin and ethylene (Muday et al., 2012). To our knowledge there have been no studies to date that characterize ethylene function in nectaries, although few do show ethylene-related genes present in nectary tissues (Tang et al., 1994) or ethylene production with *CRC* promoters (Switzenberg et al., 2015). Our transcriptome has multiple ethylene-related genes that are expressed across all developmental stages, e.g., *CvEIN4* (*ETHYLENE INSENSITIVE 4*) and *CvEIL3* (*ETHYLENE INSENSITIVE-LIKE 3*) (**Figure 6C**). Further, *CHITINASE LIKE 1* (*CLK1*), which modulates ethylene biosynthesis in root development, is among the highest expressed transcripts (**Table S4**). However, it is yet unclear what role these genes play, and whether they have a function unique to nectaries.

## 5 Conclusions

As no explicit ancestral reconstruction states of nectaries have been performed in Brassicales or core eudicots, it remains unknown whether nectaries in Cleomaceae and Brassicaceae represent a single or independent origin of nectaries. The data presented in this study demonstrate a high degree of conservation between Cleomaceae and Brassicaceae, which would be consistent with a single origin of nectaries in these sister lineages. *CvCRC* functions as it does in *Arabidopsis* and is regulated redundantly by MADS-box genes *AG* and *SHP*. *Cleome violacea* nectaries are eccrine-based and appear to regulate their own energy production. Given multiple origins of other traits (e.g., monosymmetry), we cannot exclude the possibility of independent recruitment in the roles of *CRC, AG, SHP* and *SWEET9* for nectary development and nectar secretion, respectively. Research on the evolution and development of nectaries and on nectar biology is ripe for interdisciplinary research (Liao et al., 2021). Here we show that *Cleome violacea* is a promising model for nectary development in the Cleomaceae that will pave the way forward for future nectary research on other key factors such as morphology and pollination.

## Data availability statement

The raw reads used in this study are available at the sequence read archive (SRA) database under BioProject ID: PRJNA912718.

## Author contributions

SC and JH conceived the study. SC collected and analysed the VIGS and transcriptomic data. AD collected nectar volume data. AD and SC prepared histological slides. BZ produced SEM micrographs. SC wrote the initial manuscript draft. SC, BZ, and JH edited the manuscript. All authors contributed to the article and approved the submitted version.

## Supplementary material

Supplementary Figure 1 ExN50 graph generated by the ‘contig_ExN50_statistic.pl’ and ‘plot_ExN50_statistic.Rscript’ scripts provided with Trinity.

Supplementary Figure 2 A heatmap of nectary-related genes displayed as log2(TPM).

Supplementary Figure 3 Sequence and ORF of uncharacterized Trinity transcript DN802_c0_g1_i4.. The ORF is highlighted and begins at bp 182.

Supplementary Figure 4 Flowers of *Cleome violacea* treated with pTRV2-DN802_c0_g1_i4-*CvANS* constructs. Phenotypes were indiscernible from pTRV2-*CvANS* control. **(A)** Flower with mild yellowing and underdeveloped stamens. **(B)** Flower with strong yellowing and no nectar production. **(C)** Flower with mild yellowing and underdeveloped stamens. **(D)** Flower with moderate yellowing. **(E)** Flower with moderate yellowing and underdeveloped stamen. **(F)** Flower with moderate yellowing. Scale bars = 1 mm.

Supplementary Figure 5 Z-score heatmaps of TransDecoder filtered transcripts; **(A)** All differentially expressed transcripts and **(B)** transcripts with TPM > 100 from Cleome violacea pre-anthetic, anthetic, and post-anthetic nectaries.
