# Supplementary figures and images for: Nectary development in Cleome violacea
Source: Front Plant Sci. 2023 Feb 9;13:1085900. doi: 10.3389/fpls.2022.1085900 (PMC9949531; doi:10.3389/fpls.2022.1085900)

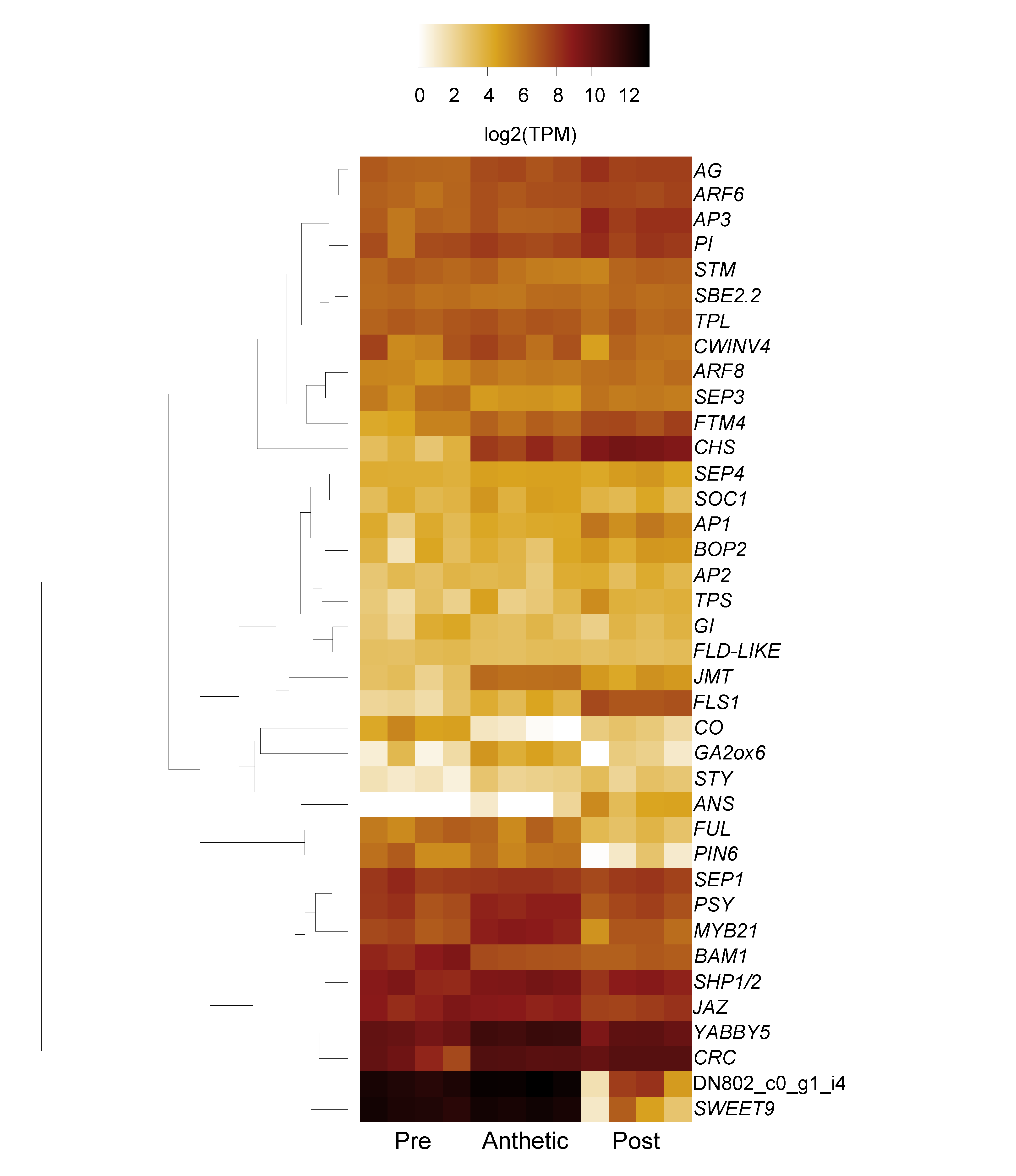

Supplement: Supplementary Figure 2 — A heatmap of nectary-related genes displayed as log2(TPM). [file Image_2.tiff]

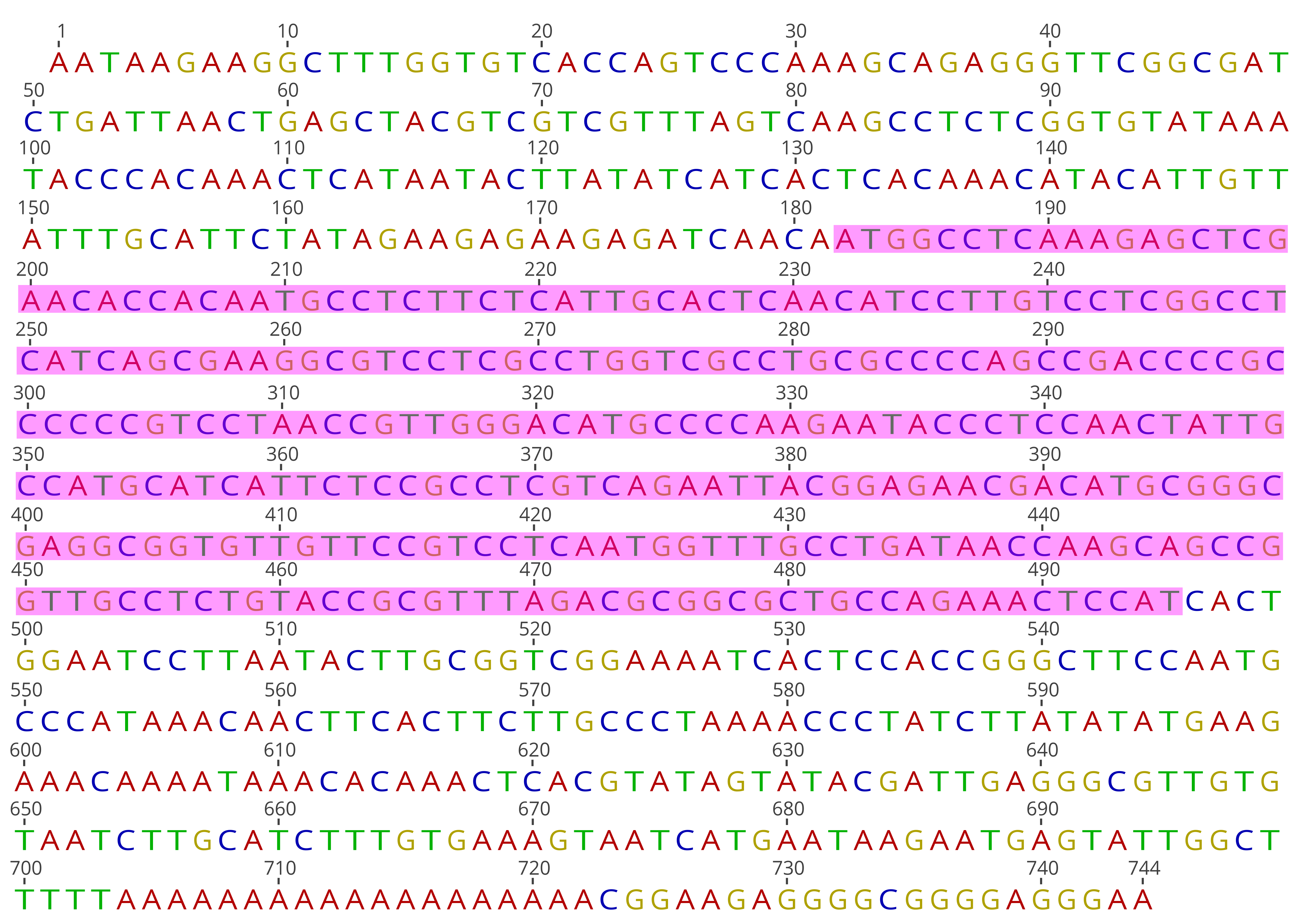

Supplement: Supplementary Figure 3 — Sequence and ORF of uncharacterized Trinity transcript DN802_c0_g1_i4.. The ORF is highlighted and begins at bp 182. [file Image_3.tiff]

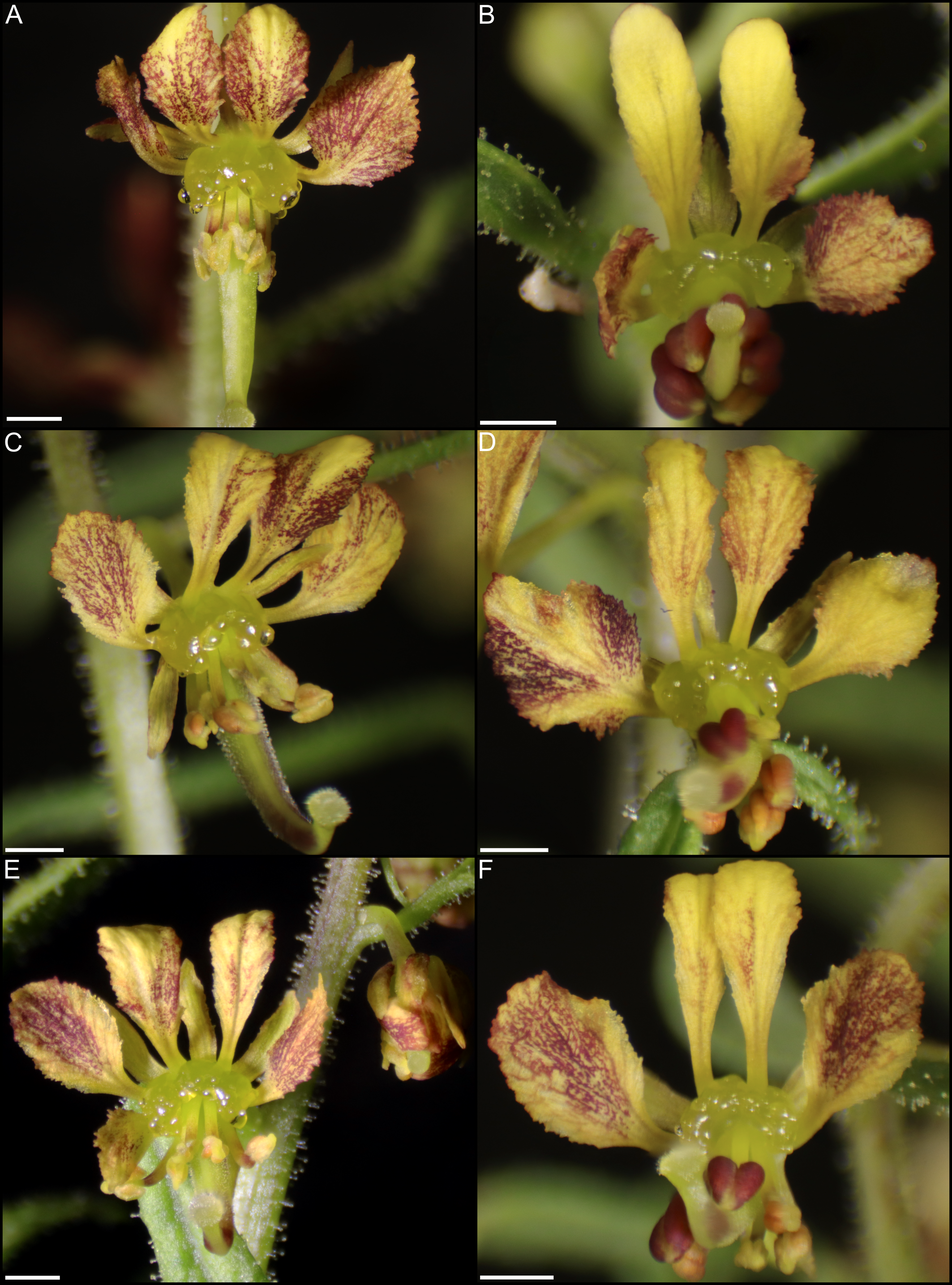

Supplement: Supplementary Figure 4 — Flowers of Cleome violacea treated with pTRV2-DN802_c0_g1_i4-CvANS constructs. Phenotypes were indiscernible from pTRV2-CvANS control. (A) Flower with mild yellowing and underdeveloped stamens. (B) Flower with strong yellowing and no nectar production. (C) Flower with mild yellowing and underdeveloped stamens. (D) Flower with moderate yellowing. (E) Flower with moderate yellowing and underdeveloped stamen. (F) Flower with moderate yellowing. Scale bars = 1 mm. [file Image_4.tiff]

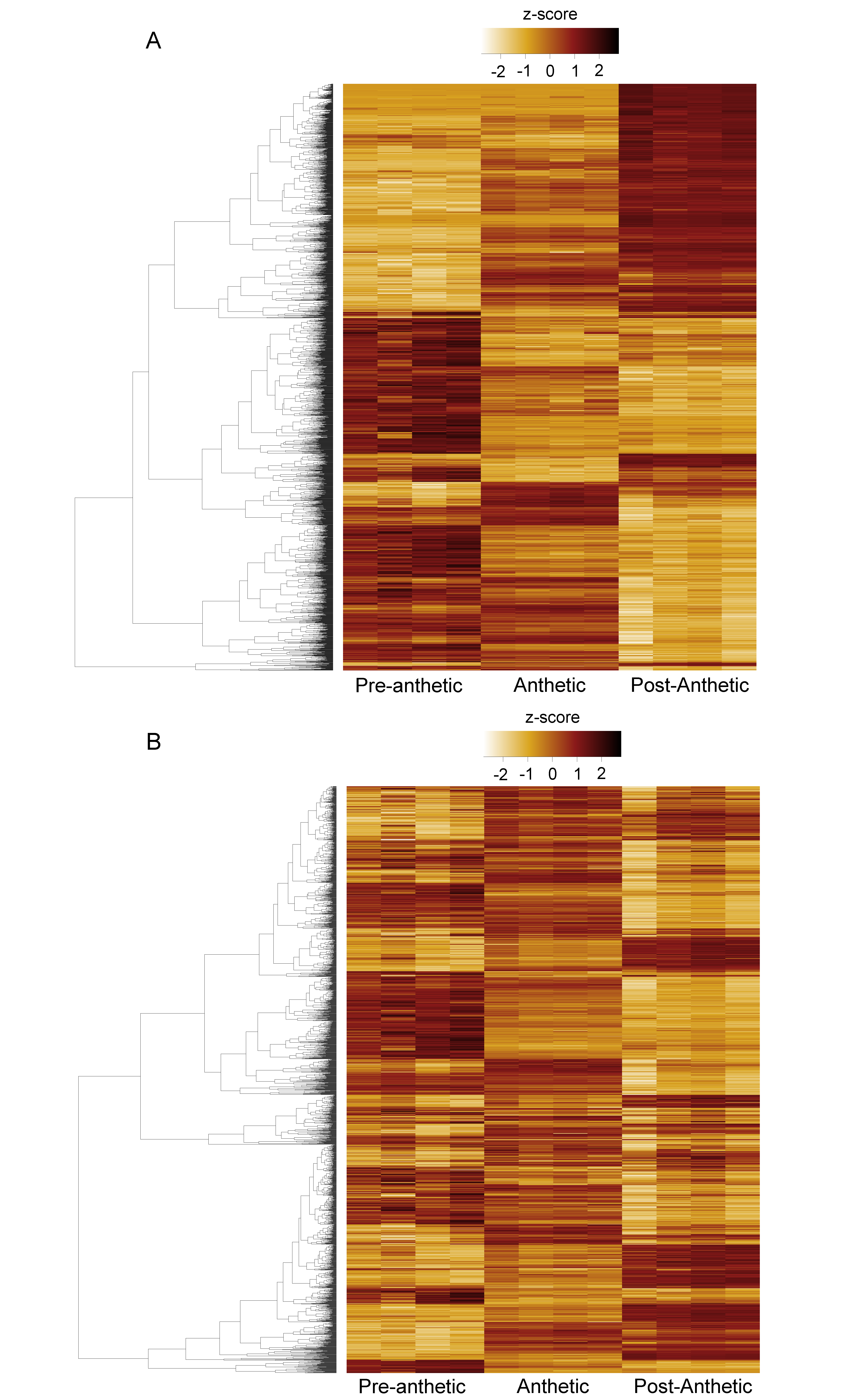

Supplement: Supplementary Figure 5 — Z-score heatmaps of TransDecoder filtered transcripts; (A) All differentially expressed transcripts and (B) transcripts with TPM > 100 from Cleome violacea pre-anthetic, anthetic, and post-anthetic nectaries. [file Image_5.tiff]
