# Supplementary material for: Nectary development in Cleome violacea
Source: Front Plant Sci. 2023 Feb 9;13:1085900. doi: 10.3389/fpls.2022.1085900 (PMC9949531; doi:10.3389/fpls.2022.1085900)
Supplement: Supplementary file 6 [file DataSheet_1.docx]

**Table S1.** Software versions and references for programs used in this study.

| Software | Version | References |
| --- | --- | --- |
| Trim Galore | 0.6.6 | Krueger, 2012 |
| FastQC | 0.11.9 | Andrews, 2010 |
| Trinity | 2.12 | Haas et al., 2013 |
| Corset | 1.09 | Davidson and Oshlack, 2014 |
| edgeR | 3.32.1 | Robinson et al., 2009 |
| R | 4.0.5 | R Core Team, 2013 |
| BLAST+ | 2.2.31 | Altschul et al., 1990 |
| Geneious | 11.09 | Geneious 11.09 (https://www.geneious.com) |
| SiFi21 | 1.2.3 | Lück et al., 2019 |
| BUSCO | 5.1.2 | Simão et al., 2015 |
| Transdecoder | 5.0.1 | https://github.com/TransDecoder |

Table S1 References.

Altschul, S. F., Gish, W., Miller, W., Myers, E. W., & Lipman, D. J. (1990). Basic local alignment search tool. *Journal of molecular biology*, *215*(3), 403-410.

Andrews, S. (2010). FastQC: a quality control tool for high throughput sequence data.

Davidson, N. M., & Oshlack, A. (2014). Corset: enabling differential gene expression analysis for de novo assembled transcriptomes. *Genome biology*, *15*(7), 1-14.

Haas, B. J., Papanicolaou, A., Yassour, M., Grabherr, M., Blood, P. D., Bowden, J., ... & Regev, A. (2013). De novo transcript sequence reconstruction from RNA-seq using the Trinity platform for reference generation and analysis. *Nature protocols*, *8*(8), 1494-1512.

Krueger, F. (2012). Trim Galore: a wrapper tool around Cutadapt and FastQC to consistently apply quality and adapter trimming to FastQ files, with some extra functionality for MspI-digested RRBS-type (Reduced Representation Bisufite-Seq) libraries. Available at: *http://www. bioinformatics. babraham. ac. uk/projects/trim_galore/.*

Lück, S., Kreszies, T., Strickert, M., Schweizer, P., Kuhlmann, M., & Douchkov, D. (2019). siRNA-Finder (si-Fi) software for RNAi-target design and off-target prediction. *Frontiers in Plant Science*, *10*, 1023.

R Core Team (2013). R: A language and environment for statistical computing (Vienna, Austria: R Foundation for Statistical Computing). Available at: http:// www.r-project.org/.

Robinson, M. D., McCarthy, D. J., & Smyth, G. K. (2010). edgeR: a Bioconductor package for differential expression analysis of digital gene expression data. *bioinformatics*, *26*(1), 139-140.

Simão, F. A., Waterhouse, R. M., Ioannidis, P., Kriventseva, E. V., & Zdobnov, E. M. (2015). BUSCO: assessing genome assembly and annotation completeness with single-copy orthologs. *Bioinformatics*, *31*(19), 3210-3212.

**Table S2.** Automatically generated Trinity assembly statistics.

|  | **All Isoforms** | **Longest Isoform** |
| --- | --- | --- |
| Contig N10 | 5247 | 4641 |
| Contig N20 | 4101 | 3584 |
| Contig N30 | 3424 | 2945 |
| Contig N40 | 2936 | 2472 |
| Contig N50 | 2541 | 2032 |
| Median contig length | 991 | 368 |
| Average contig | 1478.87 | 865.39 |
| Total assembled bases | 212837382 | 62520918 |
| Total trinity 'genes': | 72246 | |
| Total trinity transcripts: | 143919 | |
| Percent GC | 43.71 | |

Table S3. BUSCO analysis using version 5.1.2 with the Viridiplantae_odb10 database. Analysis was run in transcriptome mode using hmmsearch version 3.1 and metaeuk version 6.a5d39d9.

| Complete BUSCOs (C) | 423 |
| --- | --- |
| Complete and single-copy BUSCOs (S) | 44 |
| Complete and duplicated BUSCOs (D) | 379 |
| Fragmented BUSCOs (F) | 2 |
| Missing BUSCOs (M) | 0 |
| Total BUSCO groups Searched (n) | 425 |
| C:99.6%[S:10.4%,D:89.2%],F:0.4%,M:0%,n:425 | |

**Table S4.** Highest expressed transcripts in the *Cleome violacea* nectary transcriptome (n = 51); transcripts were taken from TransDecoder filtered lists of all significantly differentially expressed transcripts and overall highest TPM transcripts.

| **Name** | **Accession** | **Related Function of Homologues** | **Potential Role in *Cleome violacea* Nectaries** | **Citation** |
| --- | --- | --- | --- | --- |
| *BGLU19* | AT3G21370 | Disease resistance in rice | Biotic stress | Li et al., 2019 |
| *APX1* | AT1G07890 | H2O2 scavenging | Biotic stress | Pnueli et al., 2003 |
| *LTP2* | AT2G38530 | Enhanced tolerance to *Pseudomonas* | Biotic stress | Molina and Olmedo 1997 |
| *SAG21* | AT4G02380 | Root development and biotic stress | Biotic stress | Salleh et al., 2012 |
| *GAMMA-VPE* | AT4G32940 | Resistance to *Botrytis* | Biotic stress | Baarlen et al., 2007 |
| *AT4G30170* | AT4G30170 | Oxidative stress response in sulfate-transporter mutant | Abiotic stress | Maruyama-Nakashita et al., 2003 |
| *CHS* | AT5G13930 | Accumulation of flavonoids and abiotic/biotic resistance | Abiotic/biotic stress | Dao et al.,2011 |
| *AT5G38100* | AT5G38100 | General response to water stress | Water stress | Chen et al., 2003 |
| *FER1* | AT5G01600 | Protection against oxidative stress | Abiotic stress | Ravet et al., 2009 |
| *ASMT* | AT4G35160 | Drought tolerance | Water stress | Zuo et al., 2014 |
| *ERD14* | AT1G76180 | Dehydration stress | Water stress | Kiyosue et al., 1994 |
| *HTA12* | AT5G02560 | Stomatal closure | Nectar secretion via nectarostomata | Xu et al., 2016 |
| *SPDS1* | AT1G23820 | Water stress | Water stress | Alcazar et al., 2006 |
| *AGT3* | AT2G38400 | Salt/ABA related water stress | Water stress | Bray 2004 |
| *AT1G62480* | AT1G62480 | Signal transduction related to NaCl stress | Water stress | Jiang et al., 2007 |
| *AT1G11910.2* | AT1G11910 | Draught tolerance | Water stress | Fernando et al., 2020 |
| *AT5G10770* | AT5G10770 | Draught and salinity stress | Water stress | Shariatipour and Heidari et al., 2018 |
| *PSBR* | AT1G79040 | Photosystem II complex | Energy metabolism | Allahverdiyeva et al., 2007 |
| *ELIP1* | AT3G22840 | Photoprotection | Energy metabolism | Casazza et al., 2005 |
| *LHB1B1* | AT2G34430 | Photosystem II binding protein | Energy metabolism | Cheminant et al., 2011 |
| *LHCB2.1* | AT2G05100 | Photosystem II binding protein | Energy metabolism | Cheminant et al., 2011 |
| *FBN1B* | AT4G22240 | Thylakoid maintenance | Energy metabolism | Gámez-Arjona et al., 2014 |
| *YAB5* | AT2G26580 | Can dimerize with CRC | Interacts with CRC | Gross et al., 2018 |
| *GAPC1* | AT3G04120 | Glycolysis/carbon flux/mitochondrial function | Energy metabolism | Rius et al., 2008 |
| *AT5G54940.3* | AT5G54940 | Expressed in root hair | Unclear | Li and Lan 2015 |
| *AILP1* | AT5G19140 | Expressed in shoot meristem/aluminium ion response | Unclear | Yadav et al., 2014 |
| *AT1G78040.3* | AT1G78040 | Functionally uncharacterized extension family member | Unclear | Luo et al., 2012 |
| *UBQ3* | AT5G03240 | Protein degradation | Unclear | Sun and Callis 1997 |
| *TUA2* | AT1G50010 | Ubiquitous / many roles | Unclear | Abe et al., 2004 |
| *TUA4* | AT1G04820 | Ubiquitous / many roles | Unclear | Abe et al., 2004 |
| *AT2G20870* | AT2G20870 | Cell wall protein downregulated in *ft* mutants | Unclear | Cai et al., 2007 |
| *VAT1* | AT5G16290 | BCAA synthesis / accumulation | Nectar composition | Chen et al., 2010 |
| *TP2* | AT1G07340 | High-affinity monosaccharide transporter | Nectar composition | Schneidereit et al., 2003 |
| *CWINV1* | AT3G13790 | CWINV4 is required for nectar production | Nectar production | Ruhlmann et al., 2010 |
| *SUSY1* | AT5G20830 | Highly expressed in *Nicotiana* floral nectaries | Nectar production | Ren et al., 2007 |
| *PIP2;5* | AT3G54820 | Aquaporin in *Aquilegia* | Nectar production | Singh et al., 2020 |
| *PIP1A* | AT3G61430 | Aquaporin in *Aquilegia* | Nectar production | Singh et al., 2020 |
| *GAMMA-TIP* | AT2G36830 | Aquaporin in *Aquilegia* | Nectar production | Singh et al., 2020 |
| *PIP2;2* | AT2G37170 | Aquaporin in *Aquilegia* | Nectar production | Singh et al., 2020 |
| *DELTA-TIP* | AT3G16240 | Aquaporin in *Aquilegia* | Nectar production | Singh et al., 2020 |
| *BGLU20* | AT1G75940 | Anther Specific Glucosidase | Glucose production | Rubinelli et al., 1998 |
| *SWEET9* | AT2G39060 | Nectar Production | Nectar production | Lin et al., 2014 |
| *ACP2* | AT1G54580 | Increased expression in presence of sucrose | Nectar composition | Bonaventure and Ohlrogge 2002 |
| *CASPL1E2* | AT4G15620 | Bind with *GAI* | Unclear | Barro-Trastoy et al., 2022 |
| *SIP3* | AT4G30960 | Aquaporin | Nectar production | Quigley et al., 2001 |
| *ATBBE26* | AT5G44400 | Cell wall related in lateral root development | Growth and development | Xun et al., 2020 |
| *XTH24* | AT4G30270 | Turgor-driven polar cell elongation | Growth and development | Lee et al., 2017 |
| *EXL2* | AT5G64260 | Suppresses brassinosteroid-dependent growth and controls C allocation | Growth and development | Schröder et al., 2012 |
| *SAMDC* | AT3G02470 | Production of intermediates in the polyamine biosynthetic pathway | Growth and development | Ge et al., 2006 |
| *CLK1* | AT1G05850 | Modulates ethylene biosynthesis to regulate root development | Growth and development | Gu et al., 2019 |
| *PRX44* | AT4G26010 | Root hair growth | Growth and development | Marzol et al., 2022 |

Table S4**.** References

Abe, T., Thitamadee, S., & Hashimoto, T. (2004). Microtubule defects and cell morphogenesis in the lefty1lefty2 tubulin mutant of Arabidopsis thaliana. *Plant and cell physiology*, *45*(2), 211-220.

Alcázar, R., Cuevas, J. C., Patron, M., Altabella, T., & Tiburcio, A. F. (2006). Abscisic acid modulates polyamine metabolism under water stress in Arabidopsis thaliana. *Physiologia Plantarum*, *128*(3), 448-455.

Allahverdiyeva, Y., Mamedov, F., Suorsa, M., Styring, S., Vass, I., & Aro, E. M. (2007). Insights into the function of PsbR protein in Arabidopsis thaliana. *Biochimica et Biophysica Acta (BBA)-Bioenergetics*, *1767*(6), 677-685.

Barro‐Trastoy, D., Gomez, M. D., Blanco‐Touriñán, N., Tornero, P., & Perez‐Amador, M. A. (2022). Gibberellins regulate ovule number through a DELLA–CUC2 complex in Arabidopsis. *The Plant Journal*, *110*(1), 43-57.

Bonaventure, G., & Ohlrogge, J. B. (2002). Differential regulation of mRNA levels of acyl carrier protein isoforms in Arabidopsis. *Plant physiology*, *128*(1), 223-235.

Bray, E. A. (2004). Genes commonly regulated by water-deficit stress in Arabidopsis thaliana. *Journal of experimental botany*, *55*(407), 2331-2341.

Cai, X., Ballif, J., Endo, S., Davis, E., Liang, M., Chen, D., ... & Wu, Y. (2007). A putative CCAAT-binding transcription factor is a regulator of flowering timing in Arabidopsis. *Plant Physiology*, *145*(1), 98-105.

Casazza, A. P., Rossini, S., Rosso, M. G., & Soave, C. (2005). Mutational and expression analysis of ELIP1 and ELIP2 in Arabidopsis thaliana. *Plant molecular biology*, *58*(1), 41-51.

Cheminant, S., Wild, M., Bouvier, F., Pelletier, S., Renou, J. P., Erhardt, M., ... & Achard, P. (2011). DELLAs regulate chlorophyll and carotenoid biosynthesis to prevent photooxidative damage during seedling deetiolation in Arabidopsis. *The Plant Cell*, *23*(5), 1849-1860.

Chen, F., D'Auria, J. C., Tholl, D., Ross, J. R., Gershenzon, J., Noel, J. P., & Pichersky, E. (2003). An Arabidopsis thaliana gene for methylsalicylate biosynthesis, identified by a biochemical genomics approach, has a role in defense. *The Plant Journal*, *36*(5), 577-588.

Chen, H., Saksa, K., Zhao, F., Qiu, J., & Xiong, L. (2010). Genetic analysis of pathway regulation for enhancing branched‐chain amino acid biosynthesis in plants. *The Plant Journal*, *63*(4), 573-583.

Dao, T. T. H., Linthorst, H. J. M., & Verpoorte, R. (2011). Chalcone synthase and its functions in plant resistance. *Phytochemistry Reviews*, *10*(3), 397-412.

Gámez-Arjona, F. M., de la Concepción, J. C., Raynaud, S., & Mérida, Á. (2014). Arabidopsis thaliana plastoglobule-associated fibrillin 1a interacts with fibrillin 1b in vivo. *FEBS letters*, *588*(17), 2800-2804.

Ge, C., Cui, X., Wang, Y., Hu, Y., Fu, Z., Zhang, D., ... & Li, J. (2006). BUD2, encoding an S-adenosylmethionine decarboxylase, is required for Arabidopsis growth and development. *Cell research*, *16*(5), 446-456.

Gross, T., Broholm, S., & Becker, A. (2018). CRABS CLAW acts as a bifunctional transcription factor in flower development. *Frontiers in Plant Science*, *9*, 835. Gu, S. Y., Wang, L. C., Cheuh, C. M., & Lo, W. S. (2019). CHITINASE like1 regulates root development of dark-grown seedlings by modulating ethylene biosynthesis in Arabidopsis thaliana. *Frontiers in plant science*, *10*, 600.

Gu, S. Y., Wang, L. C., Cheuh, C. M., & Lo, W. S. (2019). CHITINASE like1 regulates root development of dark-grown seedlings by modulating ethylene biosynthesis in Arabidopsis thaliana. *Frontiers in plant science*, *10*, 600.

Jiang, Y., Yang, B., Harris, N. S., & Deyholos, M. K. (2007). Comparative proteomic analysis of NaCl stress-responsive proteins in Arabidopsis roots. *Journal of experimental botany*, *58*(13), 3591-3607.

Kiyosue, T., Yamaguchi-Shinozaki, K., & Shinozaki, K. (1994). Characterization of two cDNAs (ERD10 and ERD14) corresponding to genes that respond rapidly to dehydration stress in Arabidopsis thaliana. *Plant and Cell Physiology*, *35*(2), 225-231

Lee, Y. K., Rhee, J. Y., Lee, S. H., Chung, G. C., Park, S. J., Segami, S., ... & Choi, G. (2018). Functionally redundant LNG3 and LNG4 genes regulate turgor-driven polar cell elongation through activation of XTH17 and XTH24. *Plant Molecular Biology*, *97*(1), 23-36.

Li, B. B., Liu, Y. G., Tao, W. U., Wang, J. P., Xie, G. R., Chu, Z. H., & Ding, X. H. (2019). OsBGLU19 and OsBGLU23 regulate disease resistance to bacterial leaf streak in rice. *Journal of Integrative Agriculture*, *18*(6), 1199-1210.

Li, W., & Lan, P. (2015). Re-analysis of RNA-seq transcriptome data reveals new aspects of gene activity in Arabidopsis root hairs. *Frontiers in plant science*, *6*, 421.

Lin, I. W., Sosso, D., Chen, L. Q., Gase, K., Kim, S. G., Kessler, D., ... & Frommer, W. B. (2014). Nectar secretion requires sucrose phosphate synthases and the sugar transporter SWEET9. *Nature*, *508*(7497), 546-549.

Luo, G., Gu, H., Liu, J., & Qu, L. J. (2012). Four closely‐related RING‐type E3 ligases, APD1–4, are involved in pollen mitosis II regulation in Arabidopsis. *Journal of integrative plant biology*, *54*(10), 814-827.

Maruyama-Nakashita, A., Inoue, E., Watanabe-Takahashi, A., Yamaya, T., & Takahashi, H. (2003). Transcriptome profiling of sulfur-responsive genes in Arabidopsis reveals global effects of sulfur nutrition on multiple metabolic pathways. *Plant Physiology*, *132*(2), 597-605.

Marzol, E., Borassi, C., Carignani Sardoy, M., Ranocha, P., Aptekmann, A. A., Bringas, M., ... & Estevez, J. M. (2022). Class III Peroxidases PRX01, PRX44, and PRX73 Control Root Hair Growth in Arabidopsis thaliana. *International Journal of Molecular Sciences*, *23*(10), 5375.

Molina, A., Goy, P. A., Fraile, A., Sánchez-Monge, R., & García-Olmedo, F. (1993). Inhibition of bacterial and fungal plant pathogens by thionins of types I and II. *Plant Science*, *92*(2), 169-177.

Molina, A., & García‐Olmedo, F. (1997). Enhanced tolerance to bacterial pathogens caused by the transgenic expression of barley lipid transfer protein LTP2. *The Plant Journal*, *12*(3), 669-675.

Pnueli, L., Liang, H., Rozenberg, M., & Mittler, R. (2003). Growth suppression, altered stomatal responses, and augmented induction of heat shock proteins in cytosolic ascorbate peroxidase (Apx1)‐deficient Arabidopsis plants. *The Plant Journal*, *34*(2), 187-203.

Quigley, F., Rosenberg, J. M., Shachar-Hill, Y., & Bohnert, H. J. (2001). From genome to function: the Arabidopsis aquaporins. *Genome biology*, *3*(1), 1-17.

Ravet, K., Touraine, B., Boucherez, J., Briat, J. F., Gaymard, F., & Cellier, F. (2009). Ferritins control interaction between iron homeostasis and oxidative stress in Arabidopsis. *The Plant Journal*, *57*(3), 400-412.

Ren, G., Healy, R. A., Horner, H. T., James, M. G., & Thornburg, R. W. (2007). Expression of starch metabolic genes in the developing nectaries of ornamental tobacco plants. *Plant Science*, *173*(6), 621-637.

Rius, S. P., Casati, P., Iglesias, A. A., & Gomez-Casati, D. F. (2008). Characterization of Arabidopsis lines deficient in GAPC-1, a cytosolic NAD-dependent glyceraldehyde-3-phosphate dehydrogenase. *Plant physiology*, *148*(3), 1655-1667.

Rubinelli, P., Hu, Y., & Ma, H. (1998). Identification, sequence analysis and expression studies of novel anther-specific genes of Arabidopsis thaliana. *Plant molecular biology*, *37*(4), 607-619.

Ruhlmann, J. M., Kram, B. W., & Carter, C. J. (2010). CELL WALL INVERTASE 4 is required for nectar production in Arabidopsis. *Journal of experimental botany*, *61*(2), 395-404.

Salleh, F. M., Evans, K., Goodall, B., Machin, H., Mowla, S. B., Mur, L. A., ... & Rogers, H. J. (2012). A novel function for a redox‐related LEA protein (SAG21/AtLEA5) in root development and biotic stress responses. *Plant, cell & environment*, *35*(2), 418-429.

Schneidereit, A., Scholz-Starke, J., & Buttner, M. (2003). Functional characterization and expression analyses of the glucose-specific AtSTP9 monosaccharide transporter in pollen of Arabidopsis. *Plant Physiology*, *133*(1), 182-190.

Schröder, F., Lisso, J., & Müssig, C. (2012). Expression pattern and putative function of EXL1 and homologous genes in Arabidopsis. *Plant signaling & behavior*, *7*(1), 22-27.

Sebastián D, Fernando FD, Raúl DG, Gabriela GM. (2020). Overexpression of Arabidopsis aspartic protease APA1 gene confers drought tolerance. *Plant Science*, *292*, 110406.

Shariatipour, N., & Heidari, B. (2018). Investigation of Drought and Salinity Tolerance Related Genes and their Regulatory Mechanisms in Arabidopsis. *The Open Bioinformatics Journal*, *11*, 12-28.

Singh, S., Bhatt, V., Kumar, V., Kumawat, S., Khatri, P., Singla, P., ... & Sonah, H. (2020). Evolutionary understanding of aquaporin transport system in the basal eudicot model species Aquilegia coerulea. *Plants*, *9*(6), 799.

Sun, C. W., & Callis, J. (1997). Independent modulation of Arabidopsis thaliana polyubiquitin mRNAs in different organs and in response to environmental changes. *The Plant Journal*, *11*(5), 1017-1027.

van Baarlen, P., Woltering, E. J., Staats, M., & van Kan, J. A. (2007). Histochemical and genetic analysis of host and non‐host interactions of Arabidopsis with three Botrytis species: an important role for cell death control. *Molecular Plant Pathology*, *8*(1), 41-54.

Wang, C., Ding, Y., Yao, J., Zhang, Y., Sun, Y., Colee, J., & Mou, Z. (2015). Arabidopsis Elongator subunit 2 positively contributes to resistance to the necrotrophic fungal pathogens Botrytis cinerea and Alternaria brassicicola. *The Plant Journal*, *83*(6), 1019-1033.

Xu, W., Li, Y., Cheng, Z., Xia, G., & Wang, M. (2016). A wheat histone variant gene TaH2A. 7 enhances drought tolerance and promotes stomatal closure in Arabidopsis. *Plant cell reports*, *35*(9), 1853-1862.

Xun, Q., Wu, Y., Li, H., Chang, J., Ou, Y., He, K., ... & Li, J. (2020). Two receptor‐like protein kinases, MUSTACHES and MUSTACHES‐LIKE, regulate lateral root development in Arabidopsis thaliana. *New Phytologist*, *227*(4), 1157-1173.

Yadav, R. K., Tavakkoli, M., Xie, M., Girke, T., & Reddy, G. V. (2014). A high-resolution gene expression map of the Arabidopsis shoot meristem stem cell niche. *Development*, *141*(13), 2735-2744.

Zuo, B., Zheng, X., He, P., Wang, L., Lei, Q., Feng, C., ... & Kong, J. (2014). Overexpression of MzASMT improves melatonin production and enhances drought tolerance in transgenic Arabidopsis thaliana plants. *Journal of Pineal Research*, *57*(4), 408-417.

**Table S5.** (A-V) Tables generated from the KEGG automated annotation server using BLAST and bi-directional best hits with the *Arabidopsis* *thaliana*, *Brassica* *rapa* and *Tarenaya* *hassleriana* gene data sets. Transcripts lists for each stage had TPM expression above 10 and a coefficient of variation less than 50. Bolding indicates differences between stages that are greater than 3. Categories with less than 3 were removed. S1 = bud pre-anthesis; S2 = flower at anthesis; S3 senescent flower with ~10mm gynoecium; B. = Biosynthesis; D. Degradation. M. = Metabolism; P. = Pathway.

| **(A) Carbohydrate Metabolism** | **ID** | **S1** | **S2** | **S3** |
| --- | --- | --- | --- | --- |
| Glycolysis | 10 | 31 | 34 | 33 |
| Amino/Nucl sugar M. | 520 | 32 | 32 | 34 |
| Pyruvate M. | 620 | 30 | 32 | 32 |
| Starch/sucrose M. | 500 | 30 | 30 | 31 |
| Inositol phosphate M. | 562 | 25 | 26 | 26 |
| Glyoxylate M. | 630 | 26 | 25 | 25 |
| Citrate cycle | 20 | 19 | 19 | 19 |
| Propanoate M. | 640 | 18 | 19 | 19 |
| Ascorbate/aldarate M. | 53 | 17 | 19 | 17 |
| Fructose/mannose M. | 51 | 16 | 16 | 17 |
| Pentose phosphate P. | 30 | 16 | 16 | 16 |
| Galactose M. | 52 | 13 | 14 | 14 |
| Pentose interconversions | 40 | 13 | 12 | 12 |
| Butanoate M. | 650 | 12 | 12 | 12 |
| C5-dibasic acid M. | 660 | 5 | 5 | 5 |

| **(B) Amino Acid Metabolism** | **ID** | **S1** | **S2** | **S3** |
| --- | --- | --- | --- | --- |
| Cysteine/methionine M. | 270 | 38 | 40 | 41 |
| Glycine, serine/threonine M. | 260 | 29 | 30 | 29 |
| Alanine, aspartate/glutamate M. | 250 | 27 | 27 | 27 |
| Valine, leucine/isoleucine D. | 280 | 22 | 23 | 22 |
| Arginine/proline M. | 330 | 22 | 23 | 22 |
| Phenylalanine, tyrosine/tryptophan B. | 400 | 22 | 22 | 23 |
| Arginine B. | 220 | 20 | 20 | 20 |
| Tryptophan M. | 380 | 14 | 17 | 16 |
| **Tyrosine M.** | 350 | 10 | 13 | 14 |
| **Phenylalanine M.** | 360 | 9 | 13 | 13 |
| Histidine M. | 340 | 11 | 12 | 11 |
| **Lysine D.** | 310 | 8 | 12 | 11 |
| Valine, leucine/isoleucine B. | 290 | 10 | 10 | 10 |
| Lysine B. | 300 | 7 | 7 | 8 |

| **(C) Nucleotide Metabolism** | **ID** | **S1** | **S2** | **S3** |
| --- | --- | --- | --- | --- |
| Purine M. | 230 | 40 | 40 | 43 |
| **Pyrimidine M.** | 240 | 23 | 24 | 27 |

| **(D) Metabolism of Other Amino Acids** | **ID** | **S1** | **S2** | **S3** |
| --- | --- | --- | --- | --- |
| beta-Alanine M. | 410 | 15 | 17 | 16 |
| Glutathione M. | 480 | 16 | 15 | 17 |
| Selenocompound M. | 450 | 9 | 9 | 9 |
| Cyanoamino acid M. | 460 | 8 | 7 | 9 |
| Phosphonate/phosphinate M. | 440 | 3 | 3 | 3 |

| **(E) Glycan Biosynthesis and Metabolism** | **ID** | **S1** | **S2** | **S3** |
| --- | --- | --- | --- | --- |
| N-Glycan B. | 510 | 24 | 26 | 26 |
| Various types of N-glycan B. | 513 | 19 | 20 | 21 |
| GPI-anchor B. | 563 | 12 | 13 | 13 |
| Other glycan D. | 511 | 8 | 9 | 9 |
| O-Antigen nucleotide sugar B. | 541 | 7 | 7 | 7 |
| Other types of O-glycan B. | 514 | 6 | 6 | 6 |
| Glycosaminoglycan D. | 531 | 5 | 5 | 5 |
| Glycosphingolipid B. | 603 | 3 | 3 | 3 |
| Lipopolysaccharide B. | 540 | 3 | 3 | 3 |

| **(F) Lipid Metabolism** | **ID** | **S1** | **S2** | **S3** |
| --- | --- | --- | --- | --- |
| Glycerophospholipid M. | 564 | 31 | 32 | 33 |
| **Glycerolipid M.** | 561 | 26 | 30 | 28 |
| Fatty acid B. | 61 | 16 | 16 | 16 |
| Steroid B. | 100 | 16 | 16 | 16 |
| alpha-Linolenic acid M. | 592 | 13 | 14 | 14 |
| Sphingolipid M. | 600 | 10 | 12 | 12 |
| Fatty acid D. | 71 | 10 | 11 | 11 |
| B. of unsaturated fatty acids | 1040 | 7 | 7 | 7 |
| Ether lipid M. | 565 | 6 | 6 | 6 |
| Arachidonic acid M. | 590 | 5 | 5 | 6 |
| Fatty acid elongation | 62 | 5 | 5 | 5 |
| Cutin, suberine/wax B. | 73 | 4 | 5 | 5 |
| B./D. of ketone bodies | 72 | 3 | 3 | 3 |
| Linoleic acid M. | 591 | 3 | 3 | 3 |

| **(G) Metabolism of Cofactors and Vitamins** | **ID** | **S1** | **S2** | **S3** |
| --- | --- | --- | --- | --- |
| Porphyrin/chlorophyll M. | 860 | 29 | 29 | 31 |
| **Ubiquinone B.** | 130 | 13 | 17 | 21 |
| Pantothenate/CoA B. | 770 | 14 | 15 | 16 |
| Folate B. | 790 | 14 | 13 | 14 |
| Nicotinate/nicotinamide M. | 760 | 12 | 12 | 12 |
| Thiamine M. | 730 | 9 | 10 | 10 |
| One carbon pool by folate | 670 | 9 | 10 | 10 |
| Riboflavin M. | 740 | 7 | 8 | 9 |
| Vitamin B6 M. | 750 | 7 | 7 | 7 |
| Biotin M. | 780 | 6 | 7 | 8 |
| Retinol M. | 830 | 5 | 4 | 5 |

| **(H) Xenobiotics Biodegradation and Metabolism** | **ID** | **S1** | **S2** | **S3** |
| --- | --- | --- | --- | --- |
| Drug M. - other enzymes | 983 | 12 | 13 | 15 |
| M. of xenobiotics | 980 | 4 | 3 | 4 |
| Benzoate D. | 362 | 3 | 3 | 3 |
| Styrene D. | 643 | 3 | 3 | 3 |
| Chloroalkane/ene D. | 625 | 3 | 2 | 3 |
| Drug M. - cytochrome P450 | 982 | 3 | 2 | 3 |

| **(I) Energy Metabolism** | **ID** | **S1** | **S2** | **S3** |
| --- | --- | --- | --- | --- |
| **Oxidative phosphorylation** | 190 | 35 | 25 | 25 |
| Carbon fixation | 710 | 23 | 23 | 23 |
| Methane M. | 680 | 17 | 16 | 17 |
| Sulfur M. | 920 | 13 | 14 | 14 |
| Carbon fixation in prokaryotes | 720 | 13 | 13 | 13 |
| Photosynthesis antenna proteins | 196 | 12 | 12 | 12 |
| Nitrogen M. | 910 | 10 | 8 | 9 |
| Photosynthesis | 195 | 8 | 6 | 8 |

| **(J) Metabolism of Terpenoids and Polyketides** | **ID** | **S1** | **S2** | **S3** |
| --- | --- | --- | --- | --- |
| Terpenoid backbone B. | 900 | 27 | 28 | 27 |
| Carotenoid B. | 906 | 15 | 14 | 14 |
| Brassinosteroid B. | 905 | 4 | 3 | 4 |
| Diterpenoid B. | 904 | 3 | 3 | 3 |
| Zeatin B. | 908 | 4 | 3 | 2 |
| Sesquiterpenoid/triterpenoid B. | 909 | 2 | 3 | 3 |

| **(K) Biosynthesis of other Secondary Metabolites** | **ID** | **S1** | **S2** | **S3** |
| --- | --- | --- | --- | --- |
| Phenylpropanoid B. | 940 | 18 | 20 | 21 |
| Flavonoid B. | 941 | 7 | 8 | 8 |
| Tropane B. | 960 | 7 | 8 | 8 |
| Isoquinoline alkaloid B. | 950 | 5 | 7 | 7 |
| Monobactam B. | 261 | 5 | 5 | 6 |
| Stilbenoid B. | 945 | 4 | 4 | 4 |
| Glucosinolate B. | 966 | 3 | 4 | 4 |
| Streptomycin B. | 521 | 3 | 4 | 4 |
| Prodigiosin B. | 333 | 3 | 3 | 3 |

| **(L) Transcription** | **ID** | **S1** | **S2** | **S3** |
| --- | --- | --- | --- | --- |
| Spliceosome | 3040 | 70 | 70 | 69 |
| Basal transcription | 3022 | 22 | 21 | 22 |
| RNA polymerase | 3020 | 12 | 12 | 12 |

| **(M) Translation** | **ID** | **S1** | **S2** | **S3** |
| --- | --- | --- | --- | --- |
| **Nucleocytoplasmic transport** | 3013 | 81 | 78 | 84 |
| mRNA surveillance | 3015 | 46 | 46 | 46 |
| **Ribosome biogenesis** | 3008 | 41 | 39 | 43 |
| Ribosome | 3010 | 32 | 31 | 32 |
| Aminoacyl-tRNA B. | 970 | 25 | 26 | 26 |

| **(N) Replication and Repair** | **ID** | **S1** | **S2** | **S3** |
| --- | --- | --- | --- | --- |
| **Nucleotide excision repair** | 3420 | 25 | 22 | 30 |
| **DNA replication** | 3030 | 12 | 9 | 27 |
| **Base excision repair** | 3410 | 13 | 13 | 20 |
| **Homologous recombination** | 3440 | 12 | 10 | 19 |
| **Mismatch repair** | 3430 | 9 | 6 | 13 |
| Non-homologous end-joining | 3450 | 5 | 5 | 7 |

| **(O) Environmental Adaptation** | **ID** | **S1** | **S2** | **S3** |
| --- | --- | --- | --- | --- |
| **Thermogenesis** | 4714 | 40 | 32 | 32 |
| Plant-pathogen interaction | 4626 | 26 | 25 | 26 |
| Circadian rhythm - plant | 4712 | 17 | 17 | 18 |

| **(P) Cellular Community - Prokaryotes/Eukaryotes** | **ID** | **S1** | **S2** | **S3** |
| --- | --- | --- | --- | --- |
| Quorum sensing | 2024 | 12 | 11 | 12 |
| Biofilm formation - *E*. *coli* | 2026 | 3 | 3 | 3 |
| Tight junction | 4530 | 12 | 13 | 13 |
| Focal adhesion | 4510 | 6 | 7 | 8 |
| Adherens junction | 4520 | 5 | 6 | 6 |
| Gap junction | 4540 | 4 | 4 | 5 |

| **(Q) Signal Transduction** | **ID** | **S1** | **S2** | **S3** |
| --- | --- | --- | --- | --- |
| Plant hormone signaling | 4075 | 33 | 34 | 34 |
| MAPK signaling P. - plant | 4016 | 28 | 32 | 30 |
| PI3K-Akt signaling P. | 4151 | 22 | 23 | 24 |
| mTOR signaling P. | 4150 | 23 | 22 | 24 |
| AMPK signaling P. | 4152 | 20 | 21 | 21 |
| Phosphatidylinositol signaling | 4070 | 18 | 18 | 19 |
| HIF-1 signaling P. | 4066 | 15 | 16 | 17 |
| Sphingolipid signaling P. | 4071 | 12 | 15 | 16 |
| FoxO signaling P. | 4068 | 13 | 13 | 14 |
| Wnt signaling P. | 4310 | 11 | 13 | 13 |
| Apelin signaling P. | 4371 | 12 | 12 | 13 |
| MAPK signaling P. - yeast | 4011 | 12 | 11 | 12 |
| MAPK signaling P. | 4010 | 8 | 10 | 11 |
| Two-component system | 2020 | 10 | 9 | 9 |
| Phospholipase D signaling P. | 4072 | 9 | 9 | 10 |
| TGF-beta signaling P. | 4350 | 8 | 8 | 8 |
| Calcium signaling P. | 4020 | 7 | 8 | 8 |
| Notch signaling P. | 4330 | 7 | 7 | 7 |
| Hippo signaling P. | 4390 | 7 | 7 | 7 |
| cAMP signaling P. | 4024 | 6 | 7 | 8 |
| Ras signaling P. | 4014 | 5 | 6 | 7 |
| cGMP-PKG signaling P. | 4022 | 5 | 6 | 7 |
| Hedgehog signaling P. | 4340 | 5 | 5 | 5 |

| **(R) Membrane Transport** | **ID** | **S1** | **S2** | **S3** |
| --- | --- | --- | --- | --- |
| ABC transporters | 2010 | 5 | 5 | 6 |
| Bacterial secretion system | 3070 | 5 | 5 | 5 |

| **(S) Transport and Catabolism** | **ID** | **S1** | **S2** | **S3** |
| --- | --- | --- | --- | --- |
| Autophagy - yeast | 4138 | 39 | 38 | 38 |
| Endocytosis | 4144 | 36 | 36 | 36 |
| Peroxisome | 4146 | 32 | 32 | 32 |
| Lysosome | 4142 | 26 | 27 | 26 |
| Autophagy - other | 4136 | 19 | 19 | 19 |
| Phagosome | 4145 | 15 | 16 | 16 |
| Mitophagy - yeast | 4139 | 12 | 12 | 12 |

| **(T) Folding, Sorting and Degradation** | **ID** | **S1** | **S2** | **S3** |
| --- | --- | --- | --- | --- |
| Protein processing in ER | 4141 | 63 | 63 | 62 |
| Ubiquitin proteolysis | 4120 | 34 | 34 | 37 |
| RNA D. | 3018 | 33 | 32 | 34 |
| Proteasome | 3050 | 24 | 24 | 24 |
| Protein export | 3060 | 14 | 14 | 14 |
| SNARE interactions | 4130 | 7 | 7 | 7 |
| Sulfur relay system | 4122 | 6 | 6 | 6 |

| **(U) Cell Growth and Death** | **ID** | **S1** | **S2** | **S3** |
| --- | --- | --- | --- | --- |
| **Cell cycle** | 4110 | 27 | 28 | 46 |
| **Cell cycle - yeast** | 4111 | 22 | 20 | 43 |
| **Meiosis - yeast** | 4113 | 18 | 15 | 33 |
| **Oocyte meiosis** | 4114 | 16 | 17 | 22 |
| **Cellular senescence** | 4218 | 14 | 16 | 20 |
| Necroptosis | 4217 | 11 | 11 | 11 |
| Apoptosis | 4210 | 9 | 11 | 12 |
| p53 signaling P. | 4115 | 9 | 9 | 9 |
| Ferroptosis | 4216 | 7 | 8 | 8 |
| Cell cycle - Caulobacter | 4112 | 5 | 5 | 5 |

| **(V) Cell Motility** | **ID** | **S1** | **S2** | **S3** |
| --- | --- | --- | --- | --- |
| Regulation of actin | 4810 | 9 | 10 | 11 |

**Table S6.** Phenotyping data for all VIGS treatment groups used in this study. * The *CvSHP* treatment group was from a preliminary study and used different controls.

| **Construct** | **Phenotype** | **Total Plants (#)** | **Plant Mortality (%)** | **Plants with Phenotype (%)** | **Total Altered Flowers (#)** | **Altered flowers per plant (avg.)** |
| --- | --- | --- | --- | --- | --- | --- |
| *CvCRC*+*CvANS* | Reduced or Absent Nectary + Yellow Petals | 100 | 27 | 30 | 102 | 3 |
| *CvAG*+*CvSHP* | Reduced or Absent Nectary + Increased Whorls + No stamen or carpel | 60 | 20 | 23.3 | 71 | 5 |
| DN802_c0_g1_i4+*CvANS* | N/A | 20 | 50 | 45 | 17 | 2 |
| *CvSWEET9*+*CvANS* | No nectar production | 60 | 26.7 | 21.7 | 33 | 3 |
| *CvSHP** | N/A | 46 | 23.9 | 0 | 0 | 0 |
| *CvAG* | Increased Whorls + No stamen or carpel | 20 | 25 | 35 | 69 | 10 |
| *CvANS* | Yellow Petals | 20 | 30 | 35 | 32 | 5 |
| MCS | N/A | 10 | 10 | n/a | n/a | n/a |
| UNTREATED | N/A | 10 | 0 | n/a | n/a | n/a |
